# Long-term Changes in Personal Recovery and Quality of Life Among Patients With Schizophrenia Spectrum Disorders and Different Durations of Illness: A Meta-analysis

**DOI:** 10.1093/schbul/sbae045

**Published:** 2024-04-13

**Authors:** Lars de Winter, Auke Jelsma, Jentien M Vermeulen, Jaap van Weeghel, Ilanit Hasson-Ohayon, Cornelis L Mulder, Nynke Boonstra, Wim Veling, Lieuwe de Haan

**Affiliations:** Phrenos Center of Expertise, Utrecht, the Netherlands; Department of Psychiatry, Amsterdam UMC location AMC, Amsterdam, the Netherlands; Department of Psychiatry, Amsterdam UMC location AMC, Amsterdam, the Netherlands; Department of Psychiatry, Amsterdam UMC location AMC, Amsterdam, the Netherlands; Tranzo, Tilburg University, Tilburg, the Netherlands; Department of Psychology, Bar-Ilan University, Ramat-Gan, Israel; Epidemiological and Social Psychiatric Research Institute, Erasmus Medical Center, Rotterdam, the Netherlands; Parnassia Psychiatric Institute, Rotterdam, the Netherlands; NHL Stenden University of Applied Science, Leeuwarden, the Netherlands; University Medical Center Utrecht, Division Neuroscience, Utrecht, the Netherlands; University of Groningen, University Medical Center Groningen, Groningen, the Netherlands; Department of Psychiatry, Amsterdam UMC location AMC, Amsterdam, the Netherlands

**Keywords:** subjective quality of life, changes, schizophrenia spectrum disorders, personal recovery, course, psychosis, meta-analysis

## Abstract

**Background and Hypothesis:**

In schizophrenia spectrum disorders (SSD) personal recovery and subjective quality of life (S-QOL) are crucial and show conceptual overlap. There is limited knowledge about how these outcomes change over time. Therefore, we investigated changes in personal recovery or S-QOL for patients with SSD. We specifically focused on the influence of the patients’ durations of illness (DOI) on changes in personal recovery and S-QOL.

**Study Design:**

We included 46 studies investigating longitudinal changes in quantitative assessments of personal recovery or S-QOL for patients with SSD. Outcomes were categorized in overall personal recovery, overall S-QOL connectedness, hope and optimism, identity, meaning in life, and empowerment. We evaluated effect sizes of change between baseline and follow-up assessments. We also evaluated potential moderating effects, including DOI on these changes in outcomes.

**Study Results:**

We found small improvements of overall personal recovery and S-QOL, but marginal or no improvement over time in the other more specific outcome domains. Patients without a schizophrenia diagnosis, a younger age, and more recent publications positively influenced these changes. We found no significant influence of DOI on the changes in any outcome domain.

**Conclusions:**

Improvement in personal recovery or S-QOL of people with SSD is modest at best. However, these studies did not fully capture the personal narratives or nonlinear process of recovery of an individual. Future research should focus on how to shift from a clinical to more person-oriented approach in clinical practice to support patients in improving their personal process of recovery.

**Review protocol registration:**

CRD42022377100.

## Introduction

Schizophrenia spectrum disorders (SSD)^1^ are known for affecting multiple life domains, such as social functioning, quality of life, and personal recovery.^2,3^ A dominant idea has been that SSD results in a progressive downhill course on these life domains.^4^ However, recent studies challenged these assumptions^5^ and showed favorable patterns of improvement in clinical and social recovery in SSD.^6,7^

Personal recovery and subjective quality of life (S-QOL) are crucial aspects of recovery in SSD.^8,9^ Over the years the focus of recovery has shifted from a predominantly clinical orientation toward a person-centered perspective that matches the goals and needs of patients.^9,10^ Personal recovery is defined as an ongoing personal process of adaptation and development to overcome the negative consequences of mental disorder.^11–13^ Personal recovery is a growing field of research and a major aim in recovery-oriented practice.^8,14^ It is a dynamic, nonlinear, personal process with assessments focused on subjective ratings of beliefs concerning oneself.^7,10,11^ Key processes of personal recovery are conceptualized through 5 components following the CHIME framework^14^: connectedness, hope and optimism, identity, meaning in life, and empowerment. The CHIME framework has been the most widely investigated and established framework to conceptualize personal recovery. Previous research indicated that it provides a sound theoretically based framework for clinical and research purposes.^15^

Similar to personal recovery, S-QOL also captures individual perspectives of recovery by measuring subjective components of well-being, sense of belonging, activity, self-perception, autonomy, hope, and physical health.^16^ Both constructs of S-QOL and personal recovery relate to individual reflections on personal experience, and previous studies indicated a positive association between S-QOL and personal recovery.^17,18^ Despite limited empirical support for the overlap of personal recovery and S-QOL, we observed a conceptual overlap between components of the CHIME framework of personal recovery and subdomains of S-QOL. This overlap is presented in box 1. Therefore, we decided to include both personal recovery and S-QOL in 1 meta-analysis.

**Box 1.** Overlap Between CHIME Domains of Personal Recovery and Domain of Quality of Life

CHIME Domains of Personal Recovery		Domains of Quality of Life	
Category	Definition	Category	Definition
Connectedness	The level of connectedness that an individual experiences with their social network.	Relationships and a sense of belonging	The concept of belonging, fitting with society, and the quality of relationships.
Hope and optimism about the future	The level of hope, motivation, and optimism that an individual has toward his or her future or recovery process	Hope and hopelessness	Having a positive view of the future, having goals and aspirations, and being involved in meaningful activities
Identity	The identity that an individual experiences toward themselves. This includes concepts such as different dimensions of identity, redefining a positive sense of identity, and overcoming (self-)stigma.	Self-perception	The concepts of self-efficacy, self-identity, self-stigma, and self-esteem
Meaning in life	Ratings of individuals about the meaningfulness of their lives, including the meaning of mental health experiences, spirituality, quality of life, social roles and goals, and rebuilding of their lives.	Activity	Having a feeling of being engaged into the (leisure or work) activities that people enjoy.
Empowerment	The level of empowerment and control over their own lives that individuals experience.	Autonomy, control, and choice	The level of desired dependence or independence and the sense of being in control precluded autonomy and independence.
No CHIME domain		Well-being/ Ill-being Physical health	The self-reported level of well-being including, eg, feelings of distress or problems with energy and motivation Subjective experiences and method of dealing with physical health problems.

So far, several studies investigated which factors influenced personal recovery. These studies indicated that a low level of symptoms, a younger age, higher education level, higher level of functioning, being supported by recovery-oriented practice, and being male are associated with more favorable personal recovery or S-QOL.^3,9,19–21^ Studies investigating actual changes in personal recovery and S-QOL over time for people with SSD are relatively scarce and showed mixed results regarding improvement of personal recovery.^2,22^ In other recovery domains we observed changes in positive symptoms, disorganization, and overall social functioning over time, but relatively small changes in negative symptoms, depression, and cognition (De Winter et al., manuscript submitted) were found over time.^6,7^ We also found no studies investigating the influence of illness duration (ie, the time since the first diagnosis of SSD) on changes in patients’ personal recovery. This information is crucial as processes of recovery may be different during different phases of SSD.^23,24^ More recently we found indications in meta-analyses of our group that the largest improvement in both social functioning and symptoms occurs in patients with a short duration of illness (DOI).^6,7^

Therefore, in this meta-analysis, we evaluate longitudinal changes in quantitative assessments of personal recovery or S-QOL for patients with SSD, with special focus on the influence of patients’ DOI on these changes. We specifically focused on quantitative assessments of personal recovery or S-QOL, instead of qualitative evaluations, because quantitative assessments are widely adopted in current research^8^ and are most suitable to evaluate changes in personal recovery or S-QOL. This meta-analysis is part of a comprehensive research project, including series of meta-analyses investigating changes in different recovery domains. While this meta-analysis focused on personal recovery and quality of life, previous ones were focused on social functioning, symptoms, or cognition.^6,7^ Furthermore, we investigated which factors moderated changes in personal recovery or S-QOL. We aimed to answer the following questions: (1) To what extent do personal recovery or S-QOL change over the course of SSD? (2) Which moderators at baseline are associated with changes in personal recovery or S-QOL over time?

## Methods

The meta-analysis followed PRISMA guidelines.^25^ Our protocol was preregistered in PROSPERO (CRD42022377100).

### Search Strategy

Records were identified through searches in PubMed, PsycInfo, CINAHL, and Cochrane of peer-reviewed journals until November 2023. We used terms related to the patient population (eg, schizophrenia, disorganized, paranoid, schizophreniform disorder, schizoaffective disorder, SSD, or psychosis), the study design (eg, chronicity, course, prognosis, recovery, rehabilitation, remission, decrease, decay, and longitudinal), and outcomes (eg, personal recovery, self-esteem, stigma, subjective, quality of life, or well-being) (full search strategy is reported in supplementary material 1). Additional references were traced through reference tracking of included studies and systematic reviews.

### Eligibility Criteria

Four assessors (L.d.W., K.K., R.M., and A.J.) independently selected the studies. Disagreements were resolved by consensus. The following inclusion criteria were used:

*Patient population*: Studies including adults (mean age ≥ 18) all diagnosed with schizophrenia spectrum or other psychotic disorders^26^ were included. We excluded studies describing patients with a mean age younger than 18, and studies in which patients with another classification than a schizophrenia spectrum disorder were included.
*Study design*: Longitudinal cohort study or clinical trials, with a follow-up length of at least 1 year, were included. Other study designs or studies with a shorter follow-up length were excluded.
*Outcomes*: Studies reporting self-reported uncorrected quantitative assessments of S-QOL or personal recovery (see box 1) for at least 2-time points were included. Qualitative studies and studies that only reported non-extractable data for our meta-analysis were excluded.
*Publication*: Studies published in English in peer-reviewed journals were included.

### Outcome Domains

After study selection, we categorized study outcomes into the 5 domains of the CHIME framework^14^ following the conceptual overlap between personal recovery and S-QOL described in box 1. Outcomes reporting total scores of personal recovery or S-QOL were categorized separately. This led to the following 7 outcome domains: (1) Overall personal recovery (ie, outcomes that comprise multiple CHIME domains or total scores of personal recovery assessment instruments); (2) Overall S-QOL (ie, outcomes comprising total scores of assessments of S-QOL); (3) Connectedness; (4) Hope and optimism about the future; (5) Identity; (6) Meaning in life; (7) Empowerment. An overview concerning which study outcomes are categorized in which outcome domain is described in supplementary material 2.

### Selection and Assessment of Moderators of Outcome

We selected potential moderators at baseline for study outcomes through a 3-step approach. First, we chose for a theoretically driven approach by preselecting moderators that significantly influenced personal recovery or quality of life in previous research. We executed this first selection process by extracting all significant moderators reported in at least 1 of our included studies or in reviews concerning personal recovery or S-QOL.^3,19–21,27,28^ This resulted in a long list of 79 variables to potentially take into consideration as a moderator in our meta-analysis. Second, we extracted baseline data of each of these variables: if data were available in at least 20% (ie, 9) of our included studies, we included this variable in our analysis (see Statistical analysis). The choice to exclusively select moderators that were published in at least 9 studies is in line with the recommendations of Cochrane, which suggests to exclusively execute subgroup analyses in subgroups with approximately 10 studies.^29^ This resulted in 14 potential moderators: age at baseline, antipsychotic use, DUP, education level, ethnicity, gender, baseline levels of depression, functioning, negative symptoms, overall symptoms and positive symptoms, publication year, schizophrenia diagnosis, and vocational functioning. Third, we added 6 additional moderators that were not yet established by our selection process, but deemed crucial as these were clinically and statistically relevant due to the types of studies we selected in this meta-analysis. Specifically, we added age at onset, patients’ DOI, and follow-up length of the study. Furthermore, we added baseline level of outcome, study design (clinical trial or cohort study), and whether treatment (when provided) is focused on personal recovery or S-QOL to control for potential influences of the design of our broadly included longitudinal studies. This resulted in 20 potential moderators that met our eligibility criteria (see supplementary material 4). Moderators at baseline that were evaluated by different instruments are reported in different scale levels. For these moderators, we calculated percentile scores based on normative data to ensure that they were assessed in the same scale range. We conducted an analysis of moderating effects for outcome domains that were reported by more than 9 studies (ie, 20% of our included studies): Connectedness, Meaning in life, overall personal recovery, and overall S-QOL. For these outcomes, we included moderators in the analysis if they are reported by at least 9 studies, or at least 50% of all studies reporting about this outcome domain.

### Quality Assessment

Quality assessment was conducted using the Quality in Prognostic Studies (QUIPS) tool.^30^ It was based on 6 criteria: participation, attrition, prognostic factor measurement, handling confounders, outcome measurement, and analysis and reporting. For each criterion, we assigned a high, moderate, or low risk of bias score for each study.

The first author (L.d.W.) assessed all studies and the second author (A.J.) independently conducted quality assessment of 10% of the studies. The level of agreement was substantial (κ = 0.65). Disagreements were resolved by consensus. We investigated the influence of study quality on outcomes through analyses of subgroup differences.

### Statistical Analysis

#### Meta-analytic Procedure

Meta-analyses were conducted using RevMan 5.3.^31^ We calculated effect sizes of change in study outcomes by comparing outcome assessments at baseline with each follow-up assessment within the same study. We used random effects models, weighted by the method of inverse variance.^29^ Through this method we are able to give more weight to larger studies and studies reporting effect sizes with smaller standard errors in the calculation of composite effect sizes within subgroups. For clinical trials, we analyzed longitudinal outcomes of both experimental and control groups together. Overall effect sizes of categorical outcomes were converted into Cohen’s *d*^32^ to make it similar with continuous outcomes and analyze homogeneous and consistent patterns of outcomes. We preferred the effect size of Cohen’s *d* over Hedges’ *g* because Cohen’s *d* is better suited for studies with a larger sample size of more than 20 participants, which is more common in our included studies.^33^ Magnitude of effect was considered marginal when *d* < 0.2, small when *d* ≥ 0.2 and <0.5, medium when *d* ≥ 0.5 and <0.8, and large when *d* ≥ 0.8.^32^ Statistical heterogeneity was assessed by calculating the *I*^2^ statistic (including 95% CI).^29^ We controlled for multiple testing effects in all analyses through a Benjamini-Hochberg correction, with the false discovery rate set on 0.3.^34^

#### Subgroup Analyses and Calculation of Moderators

The influence of potential moderating effects were analyzed through a meta-regression analysis using *R*.^35^ Significant moderators were further analyzed for differences in effect sizes of change between studies with high levels or presence vs studies with low levels or absence of any significant moderator, using an analysis of subgroup differences.^36^ For studies with multiple follow-up assessments, we clustered the effect size of within-person changes between baseline and each follow-up assessment into 1 composite effect size of change through the method of inverse variance.^29^

#### Handling Outliers and Publication Bias

Outliers are defined as effect sizes of individual study outcomes which confidence interval (CI) exceeded the upper or lower bound of the CI of the overall effect size of all studies. We controlled for the influence of outliers by comparing overall study outcomes with subgroups in which outliers are excluded through an analysis of subgroup differences.^36^ Potential publication bias was detected by visual inspection of funnel plots and the “trim and fill” method in which we reevaluated effect sizes after removing studies causing funnel-plot asymmetry and filling with potentially omitted studies through multiple imputations.^37^ We compared outcomes before and after the trim and fill method through an analysis of subgroup differences.^36^

## Results

### Study Flow

**Fig. 1.**
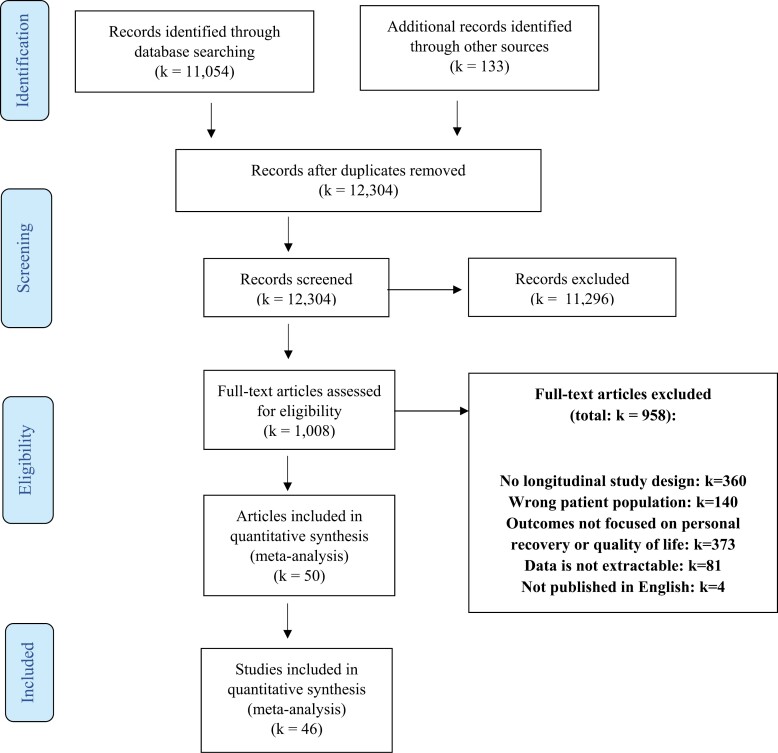
Flow chart selection studies (*k*) conform Prisma guidelines.

Of the 12 304 records primarily retrieved, we excluded 11 296 records after title and abstract screening. Of the remaining 1008 records, we excluded 958 records after full-text screening. Most of the studies were excluded during full-text selection because the study design was not longitudinal or because the outcomes did not focus on personal recovery or S-QOL (see figure 1). The remaining 50 articles reported results of 46 studies.

### Study Characteristics

The 46 included studies, describing 11 233 patients, are presented in table 1. The mean age of the total study sample was 37.1 years (*SD* = 8.6; Range = 21.3–60.3 years) and 37.9% was female. In 16 studies (34.8%), all participants were diagnosed with schizophrenia. All other studies also included patients with other SSD classifications. Twenty-five studies (54.3%) were clinical trials and 21 studies (45.7%) were cohort studies. In 10 studies (21.7%) patients received treatment that was specifically focused on improving personal recovery or S-QOL. Nine (90%) of these studies were clinical trials, and 1 (10%) was a cohort study. Twenty-one studies (45.7%) reported that all participants used antipsychotics at baseline. In 12 studies (26.1%) the mean baseline DOI was less than 5 years, in 27 studies (58.7%) at least 5 years, and in 7 studies (15.2%) unclear. In 19 studies (41.3%) the drop-out rate was low (ie, <20%), in 14 studies (30.4%) moderate (ie, ≥20% −≤40%), and in 11 studies (23.9%) high (ie, >40%). Two studies did not report drop-out rates.

**Table 1.** Descriptive Statistics of Included Studies

Study Name^a^	N (Baseline-FU)	Age (*SD*)	% Female	Primary Diagnosis	Comorbidity	Treatment	Baseline DOI (y)	FU Duration (y)	Attrition Rate	Outcome Categories Reported
Addington 2000^S1^	80-65	33.2 (8.9)	21.1%	Schizophrenia (100%)	NR	Antipsychotics (100%); Routine care (100%)	11.2 y	2.5 y	18.8%	Overall subjective quality of life
Beaudoin^S2^	919-919	41.1 (11.0)	27.1%	Schizophrenia (100%)	Obsessive-Compulsive disorder (4.4%); Other anxiety disorder (8.5%); Major depression (13.5%); Alcohol dependence (8.4%); Alcohol abuse (8.5%); Drug dependence (6.9%); Drug abuse (11.0%); Antisocial personality disorder (0.5%); Other personality disorder (1.0%); Other comorbid diagnosis (4.0%)	NR	>10 y	1.5 y	24.7%	Overall subjective quality of life
Buonocore 2018^S3^	60-60	34.9 (9.7)	45.3%	Schizophrenia (100%)	NR	Computer-assisted CRT (100%); Standard rehabilitation therapy (SRT; 100%); Risperidone (23%); Haloperidol (15%); Clozapine (39%); Olanzapine (7%); Aripiprazole (8%); Paliperidone (2%); Fluphenazine (3%); Chlorpromazine (3%)	10.8 y	5 y	6.3%	Overall subjective quality of life
Chan 2003^S4^	25-25	40.4 (7.8)	44.0%	Schizophrenia (100%)	NR	NR	15.4 y	0.3 y; 0.7 y; 1 y	16.0%	Connectedness; Meaning in life
Chien 2014^S5,S6^	124-124	25.6 (7.7)	43.0%	Schizophrenia (100%)	NR	Antipsychotics (71.0%); Antidepressants (12.1%)	2.6 y	1 y; 2 y	0.9%	Connectedness; Overall personal recovery
Conley 2007^S7^	2228-1167	41.8 (11.2)	38.5%	Schizophrenia (57.18%); Schizoaffective disorder (33.57%); Other psychotic disorder (9.25%)	Substance use disorder: 27.96%; Personality disorder: 14.50%; Depressive disorder: 39.36%	Antidepressants (38.78%); Anti-anxiety agents (11.31%); Mood stabilizers (31.24%); Hypnotics (1.71%); Antiparkinsonian agents (44.79%); Atypical antipsychotics (59.78%); Typical antipsychotics (58.21%)	21.6 y	3 y	4.3%	Connectedness; Overall subjective quality of life
Dellazizzo 2021^S8^	74-30	42.5 (12.7)	24.3%	Schizophrenia (77.0%); Schizoaffective disorder (23.0%)	NR	Atypical antipsychotics (96%); Virtual reality (VR)-assisted therapy (50%); Cognitive behavioral therapy (50%)	16.0 y	0.5 y; 1 y	16.2%	Overall subjective quality of life
Fernández-Modamio 2021^S9^	299-188	44.3 (13.4)	39.5%	Schizophrenia; Schizoaffective disorder	NR	Social Cognition Training Program (SCTP; 50.0%); Neurocognitive training (100%); Antipsychotics (100%)	22.9 y	0.5 y; 1 y	37.1%	Connectedness; Overall subjective quality of life
Fowler 2012^S10^	255-207	37.6 (11.0)	30.0%	Schizophrenia (85%); Schizoaffective disorder (13%); Delusional disorder (2%)	NR	NR	10.7 y	0.3 y; 1 y	18.2%	Identity
Fowler 2018^S11^	135-93	24.5 (7.9)	21.8%	Nonaffective psychosis (100%)	NR	Early Intervention Services (100%); Social recovery therapy (49.03%)	2.1 y	0.8 y; 1.3 y	17.4%	Hope and optimism; Empowerment; Meaning in life
Galderisi 2020^S12^	921-618	40.2 (10.7)	30.4%	Schizophrenia (100%)	Substance abuse (5.0%); Alcohol abuse (4.9%)	Antipsychotics (76.8%); Integrated treatment (26.8%)	16.2 y	4 y	32.9%	Connectedness; Empowerment; Hope and optimism; Identity
Godin 2019^S13^	770-325	32.7 (9.9)	26.0%	Schizophrenia (100%)	Anxiety disorder (37.40%); Tobacco smoking (51.56%); Cannabis use disorder (28.44%); Alcohol use disorder (20.13%)	Antipsychotics (21.17%); Antidepressants (25.71%)	10.7 y	1 y	61.4%	Meaning in life
Gorna 2008^S14^	125-125	24.7 (6.7)	37.8%	Schizophrenia (100%)	NR	NR	<2 y	1 y; 5 y	21.3%	Connectedness; Meaning in life; Overall subjective quality of life
Gumley 2022^S15^	73-54	43.0 (12.0)	49.3%	Schizophrenia spectrum disorder (100%)	NR	Empower intervention (57.5%); TAU (42.5%)	NR	0.5 y; 1 y	17.8%	Meaning in life; Overall personal Recovery
Hayhurst 2014^S16^	354-290	39.5 (11.4)	32.0%	Schizophrenia, schizoaffective, schizophreniform, or delusional disorder,	NR	Antipsychotics (100%)	11.6 y	1 y	18.1%	Overall subjective quality of life
Heering 2015^S17^	648-648	27.7 (8.0)	23.8%	Schizophrenia; Schizophreniform disorder; Schizoaffective disorder	NR	NR	4.4 y	3.3 y	42.1%	Overall subjective quality of life
Ito 2015^S18^	54-54	30.6 (10.1)	53.2%	Schizophrenia spectrum disorder (100%)	NR	Antipsychotics (100%)	2.0 y	0.5 y; 1 y; 1.5 y	53.9%	Overall subjective quality of life
Jørgensen 2015^S19^	101-94	37.5 (12.6)	53.5%	Schizophrenia (92.08%); Schizoaffective disorder (7.92%)	NR	Antipsychotics (100%); Guided self-determination intervention (49.5%)	9.8 y	0.3 y; 0.5 y; 1 y	7.9%	Connectedness; Empowerment; Hope and optimism; Overall personal recovery
Kane 2016^S20,S21^	404-404	23.1 (5.1)	27.5%	Schizophrenia (52.97%); Schizoaffective disorder, bipolar (5.94%); Schizoaffective disorder, depressive (14.11%); Schizophreniform disorder (16.58%); Brief psychotic disorder (0.50%); Psychotic disorder NOS (9.90%)	Alcohol abuse/dependence (36.39%); Cannabis abuse/dependence (35.64%)	Antipsychotics (83.42%); Personalized medication management, family psychoeducation, resilience-focused individual therapy, and supported employment and education (55.20%); Community care (44.80%)	3.7 y	0.5 y; 1 y; 1.5 y; 2 y	43.8%	Connectedness; Hope and optimism; Overall personal recovery; Overall subjective quality of life
Kelly 2009^S122^	43-43	44.1 (8.3)	27.9%	Schizophrenia (100%)	NR	Haloperidol (58,14%); Olanzapine (41,86%)	22.1 y	1 y	23.2%	Overall subjective quality of life
Kim 2019^S23^	87-87	33.6 (9.8)	49.4%	Schizophrenia (100%)	NR	Antipsychotics (100%)	8.8 y	0.5 y; 1 y	29.9%	Meaning in life
Kumazaki 2012^S24^	56-36	60.3 (6.2)	44.0%	Schizophrenia (100%)	NR	Optimal Treatment Project (OTP) interventions (100%)	27.6 y	1 y; 2 y; 3 y; 4 y; 5 y; 6 y; 12 y; 15 y	35.7%	Connectedness; Meaning in life; Overall subjective quality of life
Lasser 2005^S25^	582-582	40.9 (13.0)	33.9%	Schizophrenia (83.39%); Schizoaffective disorder (16.61%)	NR	Antipsychotics (98.79%)	NR	1 y	20.3%	Connectedness
Lee 2023^S26^	54-54	32.3 (9.3)	38.9%	Schizophrenia; Schizoaffective disorder	NR	Antipsychotics (100%)	12.7 y	14.1 y	40.7%	Overall subjective quality of life
Litman 2023^S27^	215-175	39.3 (10.8)	39.1%	Schizophrenia (100%)	NR	Antipsychotics (100%)	10.9 y	0.23 y; 0.46 y; 1 y	10.7%	Connectedness; Empowerment; Overall subjective quality of life
Liu 2023^S28^	96-76	36.3 (10.2)	55.2%	Schizophrenia/schizophreniform disorder (78.1%); Other Schizophrenia spectrum disorder (21.9%)	NR	Antipsychotics (100%)	11.3 y	2 y	20.8%	Meaning in life
Lopez-Morinigo 2022^S29^	77-28	47.7 (9.6)	46.8%	Schizophrenia spectrum disorder (100%)	NR	Metacognitive training (50.6%); Psychoeducation (49.4%); Clozapine (15.6%); Other antipsychotics (58.4%)	>5 y	0.15 y; 1 y	62.8%	Overall subjective quality of life
Marino 2015^S30^	63-15	22.2 (4.2)	36.9%	Schizophrenia (66.15%); Schizoaffective disorder (13.85%); Schizophreniform disorder (6.15%); Psychosis NOS (4.62%); Brief psychotic disorder (1.54%); No diagnosis (3.08%); Unknown (4.62%)	NR	Treatment connection program (100%)	10.2 y	2 y	69.2%	Connectedness; Overall personal recovery; Overall subjective quality of life
McNeely 2022^S31^	51-40	45.5 (13.7)	41.2%	Schizophrenia and related psychotic disorders (100%)	NR	Self-management Engaging Together (SET) for Health (100%)	21.3 y	1.1 y	21.6%	Hope and optimism about the future; Meaning in life
Moncrieff 2023^S32^	253-175	46.3 (11.8)	32.4%	Schizophrenia (68.77%); Other psychotic disorders (31.23%)	NR	Antipsychotics (100%)	>5 y	0.5 y; 1 y; 2 y	24.9%	Overall personal recovery; Overall subjective quality of life
Morrison 2018^S33^	75-60	23.6 (6.1)	42.7%	Schizophrenia, schizoaffective disorder, or delusional disorder	NR	CBT (68%); Antipsychotics (65.33%)	<2 y	0.5 y; 1 y	20.0%	Overall personal recovery; Overall subjective quality of life
Na 2016^S34^	25-25	28.2 (6.4)	48.0%	Schizophrenia (60.00%); Schizoaffective disorder (12.00%); Psychotic disorder NOS (28.00%)	NR	Antipsychotics (100%); Mind flower programs (100%)	NR	0.5 y; 1 y	4.0%	Empowerment; Identity
Neill 2022^S35^	85-43	39.7 (9.3)	71.8%	Schizophrenia (91.9%); Schizoaffective disorder (8.1%)	NR	Antipsychotics (100%)	15.7 y	0.2 y; 0.5 y; 1 y	49.4%	Overall subjective quality of life
Ortega 2020^S36^	61-61	24.1 (4.3)	24.6%	First episode Psychosis (100%)	NR	Antipsychotics (100%)	<5 y	1 y	unclear	Meaning in life
Prouteau 2005^S37^	55-55	34.3 (12.0)	34.6%	Schizophrenia (70.90%); Schizoaffective disorder (23.60%); Schizophreniform disorder (3.6%); Unspecified psychotic disorder (1.8%)	NR	Antipsychotics (100%); Integrated Psychological Treatment (100%); Antidepressants (23.64%); Mood stabilizers (20.00%); Antiparkinsonians (40.00%); Anxiolytics (3.64%)	7.4 y	0.5 y; 1 y; 1.3 y	0.0%	Empowerment
Rowland 2018^S38^	263-216	21.3 (4.9)	25.8%	Schizophrenia (84.52%); Delusional disorder (15.48%)	Substance use (70.03%)	Early Intervention Services (100%)	0.0 y	1 y	17.9%	Meaning in life
Salyers 2014^S39,S40^	116-70	47.7 (8.9)	20.7%	Schizophrenia (46.55%); Schizoaffective disorder (55.17%)	NR	Illness Management and Recovery (50.8%); Intensive Problem Solving (49.2%); Antipsychotics (100%)	NR	0.8 y; 1.5 y	40.7%	Hope and optimism; Overall personal recovery; Overall subjective quality of life
Schmidt 2017^S41^	120-120	34.3 (11.2)	43.3%	Schizophrenia (55%); Schizoaffective disorder (19.17%); Schizophreniform disorder (14.17%); Delusional disorder (5.83%); Psychotic disorder NOS (5.83%)	Substance use disorder (41.67%)	Quetiapine (100%); ACT integrated care treatment (100%)	NR	1 y	15.8%	Meaning in life
Sikira 2021^S42^	65-50	43.0 (11.1)	53.9%	Schizophrenia spectrum disorder (100%)	NR	Volunteer befriending intervention (50.8%); Antipsychotics (95.4%)	NR	0.5 y; 1 y	23.1%	Overall subjective quality of life
Tabo 2017^S43^	120-120	40.9 (10.9)	28.3%	Schizophrenia (100%)	NR	NR	16.3 y	1 y	unclear	Overall subjective quality of life
Usui 2022^S44^	59-59	22.6 (5.2)	37.0%	Schizophrenia (74.1%); Schizophreniform disorder (11.1%); Delusional disorder (3.7%); Psychotic disorder NOS (11.1%)	NR	Antipsychotics (100%)	<5 y	2.0 y	54.2%	Connectedness; Meaning in life
Veerman 2016^S45^	25-25	42.0 (10.4)	24.0%	Schizophrenia (100%)	Alcohol use (20%); Nicotine use (56%); Cocaine use (12%)	Clozapine (100%); Psychotherapy (8%)	19.6 y	1 y	19.4%	Overall subjective quality of life
Wilson-d’Almeida 2013^S46^	306-306	41.1 (10.1)	30.1%	Schizophrenia (100%)	NR	Antipsychotics (100%)	NR	0.5 y; 1 y	12.3%	Hope and optimism; Meaning in life
Wunderink 2009^S47^	125-125	26.4 (6.4)	31.2%	Schizophrenia (45.60%); Other nonaffective psychosis (54.40%)	Cannabis dependence (24%)	Antipsychotics (100%)	0.7 y	0.5 y; 1.3 y; 2 y	14.4%	Overall subjective quality of life
Xie 2005^S48,S49^	152-152	32.4 (7.2)	22.4%	Schizophrenia (70.41%); Schizoaffective disorder (29.59%)	Substance use disorder (100%); Alcohol use disorder (81.58%); Cannabis use disorder (44.74%); Cocaine use disorder (15.13%); Bipolar disorder (100%)	Integrated dual disorder treatment (100%)	12 y	0.5 y; 1 y; 1.5 y; 2 y; 2.5 y; 3 y; 4 y; 5 y; 6 y; 7 y; 8 y; 9 y; 10 y	23.1%	Connectedness; Meaning in life
Zäske 2018^S50^	48-24	32.0 (10.1)	45.8%	Schizophrenia (100%)	NR	Antipsychotics (100%)	<2 y	1 y	50.0%	Identity; Overall subjective quality of life

*Note*: DOI, duration of illness; NA, not applicable; NR, not reported; y, years.

^a^The reference list (S1–S50) of the included studies are presented in supplementary material 7.

### Meta-analysis of Changes in Personal Recovery or Quality of Life

**Fig. 2.**
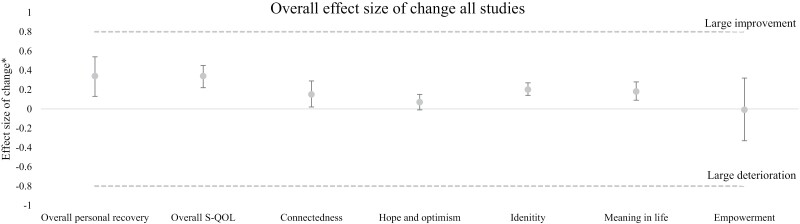
Effect sizes of change of the 7 outcome domains of quality of life and personal recovery. * In this figure a positive effect size of change indicates improvement over time and a negative effect size of change indicates deterioration over time. The upper and lower whiskers show the 95% confidence interval.

We presented an overview of the outcomes in figure 2 and table 2. In the text below *d* stands for the effect size of change, *I*^2^ stands for heterogeneity of outcomes, and *k* stands for the number of studies reporting these outcomes.

**Table 2.** Meta-analysis of Personal Recovery and Quality of Life Outcomes

Outcome Domain	*K* (Studies)	N (Baseline-FU)	Effect Size (95% CI)^a^ and Magnitude of Effect^b^	*K* Large Effect^b^ [+/−]^c^	Heterogeneity (*I*^2^ (95% CI))^a^
Overall personal recovery	8	1200-810	*d* = **0.34** [S] (0.13 to 0.54)	+ = 0/− = 1	***I*** ^**2**^ ** = 81%** (66%–89%)
Overall subjective quality of life	27	6721-4961	*d* = **0.34** [S] (0.22 to 0.45)	+ = 3/− = 0	***I*** ^**2**^ ** = 91%** (89%–93%)
Connectedness	14	5285-3517	*d* = **0.15** [N] (0.02 to 0.29)	+ = 1/− = 0	***I*** ^**2**^ ** = 95%** (94%–97%)
Hope and optimism	7	2034-1624	*d* = 0.07 [N] (−0.01 to 0.15)	+ = 0/− = 0	*I* ^2^ = 33% (4%–53%)
Identity	4	1249-874	*d* = 0.20 [S] (0.14 to 0.27)	+ = 0/− = 0	*I* ^2^ = 39% (0%–67%)
Meaning in life	15	2356-1774	*d* = **0.18** [N] (0.09 to 0.28)	+ = 0/− = 0	***I*** ^**2**^ ** = 73%** (61%–81%)
Empowerment	6	1452-1060	*d* = −0.01 [N] (−0.33 to 0.32)	+ = 0/− = 0	***I*** ^**2**^ ** = 96%** (94%–98%)

*Note:* CI, confidence interval; FU, follow-up; L, large effect; M, medium effect; N, no effect; S, small effect.

^a^Outcomes in bold are significant (*P* < .05) after Benjamini-Hochberg correction; Outcomes underlined are no longer significant after Benjamini-Hochberg correction for multiple testing.

^b^No effect (*d* > −0.20 to <0.20); Small effect (*d* ≤ −0.20 and >−0.50 to ≥0.20 and <0.50); Medium effect (*d* ≤ −0.50 and >−0.80 to ≥0.50 and <0.80); Large effect (*d* < −0.80 to >0.80).

^c^+, improvement of outcome at follow-up; −, deterioration of outcome at follow-up.

Results in our meta-analysis indicated a small improvement of *overall personal recovery* (*d* = 0.34 [0.13–0.54]; *I*^2^ = 81%; *k* = 8) and *overall S-QOL* (*d* = 0.34 [0.22–0.45]; *I*^2^ = 91%; *k* = 27). Furthermore, we found a marginal improvement of *connectedness* (*d* = 0.15 [0.02–0.29]; *I*^2^ = 95%; *k* = 14) and *meaning in life* (*d* = 0.18 [0.09–0.28]; *I*^2^ = 73%; *k* = 15). In first instance, we found a small improvement of *identity* (*d* = 0.20 [0.14–0.27]; *I*^2^ = 39%; *k* = 4). However, we found no significant improvement of identity anymore after multiple testing correction. Finally, we did not find significant changes over time in the outcome domains of *hope and optimism* (*d* = 0.07 [−0.01 to 0.15]; *I*^2^ = 33%; *k* = 7) and *empowerment* (*d* = −0.01 [−0.33 to 0.32]; *I*^2^ = 96%; *k* = 6).

### Outliers and Publication Bias

There were no outliers for hope and optimism about the future and identity. Two positive outliers and 2 negative outliers for connectedness, 1 positive and 1 negative outlier for meaning in life, 1 negative outlier for empowerment and overall personal recovery, and 5 positive and 5 negative outliers for overall S-QOL were found. We found no influence of positive or negative outliers in any of the outcome domains.

We found skewed funnel plots in Connectedness, Empowerment, and overall S-QOL (see supplementary material 6). After trimming the studies that caused asymmetry in the funnel plots and filling the funnel plot with new studies we did find small differences in effect sizes for connectedness, empowerment, and S-QOL, but none of them caused significant differences in the outcomes. Therefore, we found no indications of publication bias in our outcome domains.

### Influence of DOI on Changes in Personal Recovery or Quality of Life

Results of the analyses of moderating effects of DOI on changes in personal recovery and S-QOL is presented in supplementary material 3. Due to a lack of studies, we did not conduct an analysis of moderating effects for hope and optimism, identity, and empowerment. Meta-regression analyses indicated no significant moderating effects of the baseline DOI of the patient population on any of the investigated outcome domains.

### Analysis of Potential Moderators of Change in Outcomes at Baseline

Results of the analyses of moderating effects are presented in supplementary material 3 and table 3. Due to a lack of studies, we did not conduct an analysis of moderating effects for hope and optimism, identity, and empowerment. Furthermore, we found no moderating effects for overall personal recovery.

**Table 3.** Sensitivity Analysis of Significant Moderators

		*K* (Studies)	N (Baseline-FU)	Effect Size (95% CI)^a^ and Magnitude of Effect^b^	*K* Large Effect^b^ [+/−]^c^	Heterogeneity (*I*^2^ (95% CI))^a^
Confounder	Rating					
Overall subjective quality of life						
Baseline level of Quality of life	High level (above median)	12	3914-2586	*d* = 0.12 [N] (−0.01 to 0.24)	+ = 0/− = 0	***I*** ^**2**^ ** = 84%** (75%–89%)
	Low level (below median)	13	1673-1236	*d* = **0.57** [M] (0.35 to 0.79)	+ = 3/− = 0	***I*** ^**2**^ ** = 92%** (88%–94%)
	**Subgroup differences between follow-up cohorts**			*χ* ^*2*^ = 12.07; *df* = 1; *P* < .01		
Connectedness						
Diagnosis of schizophrenia	100% diagnosed with schizophrenia	6	1397-1034	*d* = 0.01 [N] (−0.17 to 0.20)	+ = 0/− = 0	**I** ^**2**^ ** = 86%** (71%–93%)
	<100% diagnosed with schizophrenia	7	3589-2295	*d* = **0.33** [S] (0.12 to 0.55)	+ = 1/− = 0	**I** ^**2**^ ** = 97%** (96%–98%)
	**Subgroup differences**			*χ* ^*2*^ = 5.04; *df* = 1; *P* < .05		
Meaning in life						
Age at baseline	Old age	5	508-461	*d* = −0.00 [N] (−0.12 to 0.11)	+ = 0/− = 0	*I* ^2^ = 29% (0%–55%)
	Young age	10	1848-1313	*d* = **0.27** [S] (0.16 to 0.37)	+ = 0/− = 0	***I*** ^**2**^ ** = 65%** (44%–78%)
				*χ* ^*2*^ = 11.88; *df* = 1; *P* < .01		
Baseline level of meaning in life	High	7	555-508	*d* = 0.08 [N] (−0.06 to 0.22)	+ = 0/− = 0	***I*** ^**2**^ ** = 67%** (39%–82%)
	Low	8	1801-1266	*d* = **0.27** [S] (0.12 to 0.43)	+ = 0/− = 0	***I*** ^**2**^ ** = 79%** (63%–88%)
	**Subgroup differences**			*χ* ^*2*^ = 3.40; *df* = 1; *P* = .07		
Publication year	Recent (≤10 years ago)	10	1928-1382	*d* = **0.27** [S] (0.14 to 0.40)	+ = 0/− = 0	***I*** ^**2**^ ** = 74%** (58%–84%)
	Old (>10 years ago)	4	358-338	*d* = −0.01 [N] (−0.25 to 0.23)	+ = 0/− = 0	***I*** ^**2**^ ** = 82%** (50%–93%)
	**Subgroup differences**			*χ* ^*2*^ = 4.05; *df* = 1; *P* < .05		
Diagnosis of schizophrenia	100% diagnosed with schizophrenia	6	1369-904	*d* = 0.04 [N] (−0.09 to 0.17)	+ = 0/− = 0	***I*** ^**2**^ ** = 64%** (29%–81%)
	<100% diagnosed with schizophrenia	5	670-623	*d* = **0.36** [S] (0.21 to 0.51)	+ = 0/− = 0	***I*** ^**2**^ ** = 65%** (22%–84%)
	**Subgroup differences**			*χ* ^*2*^ = 9.92; *df* = 1; *P* < .01		

*Note*: CI, confidence interval; FU, follow-up; L, large effect; M, medium effect; N, no effect; S, small effect.

^a^Outcomes in bold are significant (*P* < .05) after Benjamini-Hochberg correction; Outcomes underlined are no longer significant after Benjamini-Hochberg correction for multiple testing.

^b^No effect (*d* > −0.20 to <0.20); Small effect (*d* ≤ −0.20 and >−0.50 to ≥0.20 and <0.50); Medium effect (*d* ≤ −0.50 and >−0.80 to ≥0.50 and <0.80); Large effect (*d* < −0.80 to >0.80).

^c^+ = improvement of outcome at follow-up; − = deterioration of outcome at follow-up.

#### Moderators of Change in Overall S-QOL

Meta-regression analyses showed that baseline level of S-QOL (*B* = −0.01; *SE* = 0.01; *P* < .01; *k* = 17) significantly influenced changes in overall S-QOL. Analyses of subgroup differences indicated better improvement of overall S-QOL in studies in which patients had a low baseline level of S-QOL compared to those with a high baseline level of S-QOL (*χ*^*2*^ = 12.07; *df* = 1; *P* < .01; *k* = 25).

#### Moderators of Change in Connectedness

Meta-regression analysis showed that a schizophrenia diagnosis (*B* = 0.37; *SE* = 0.15; *P* < .05; *k* = 12) significantly influenced changes in connectedness. Analyses of subgroup differences indicated better overall improvement of connectedness in studies that also included patients with other SSD classifications compared to studies that exclusively included patients diagnosed with schizophrenia (*χ*^*2*^ = 5.04; *df* = 1; *P* < .05; *k* = 13).

#### Moderators of Change in Meaning in Life

Meta-regression analysis showed that age at baseline (*B* = −0.01; *SE* = 0.01; *P* < .05; *k* = 14), baseline level of meaning in life (*B* = −0.01; *SE* = 0.00; *P* < .05; *k* = 14), publication year (*B* = 0.02; *SE* = 0.01; *P* < .05; *k* = 14), and a schizophrenia diagnosis (*B* = 0.40; *SE* = 0.12; *P* < .05; *k* = 10) significantly influenced changes in meaning in life. Analyses of subgroup differences indicated better overall improvement of meaning in life in studies in which patients had a young age at baseline (*χ*^*2*^ = 11.88; *df* = 1; *P* < .01; *k* = 14), a more recent publication year (*χ*^*2*^ = 4.05; *df* = 1; *P* < .01; *k* = 14) and in studies that also included patients with other SSD classifications (*χ*^*2*^ = 9.92; *df* = 1; *P* < .01; *k* = 11) compared to studies with an older age, older publication years or studies exclusively including patients diagnosed with schizophrenia. The analysis of subgroup differences indicated no significant differences between studies investigating patients with a low or high baseline level of meaning in life.

### Quality Assessment

Quality assessment and the analysis of subgroup differences of QUIPS outcomes are presented in supplementary materials 4 and 5. In general study quality was relatively good concerning patient recruitment, outcome assessment, and method of analysis. A relatively large number of studies reported high risk of bias for the QUIPS items study attrition (8 studies with high ROB; handling missing data), and study confounding (15 studies with high ROB; handling confounders in the analysis).

Analysis of subgroup differences indicated that a lower study quality for study attrition positively influenced changes in S-QOL. Furthermore, a lower study quality for outcome measurement positively influenced changes in connectedness and S-QOL. A better study quality for study confounding positively influenced changes in empowerment. Finally, a better study quality for statistical analysis and reporting positively influenced changes in connectedness and S-QOL. In conclusion, lower study quality on different domains of the QUIPS tool did influence the outcomes, but we did not find a consistent line of specific QUIPS domains influencing study outcomes.

## Discussion

### Reflections on Changes in Personal Recovery and S-QOL and the Influence of DOI

In the current meta-analysis, we evaluated longitudinal changes in personal recovery or S-QOL in patients with a schizophrenia spectrum disorder with different DOI. Overall, we found a small improvement in *overall personal recovery* and *overall S-QOL*, but marginal or no improvement in the other outcome domains.

Overall, changes in personal recovery and S-QOL could be considered as modest at best, especially in comparison to more substantial improvements in other life domains such as symptoms or social functioning.^6,7^ Current findings are in line with previous research indicating that changes in quality of life and personal recovery over time are less favorable than in clinical or functional recovery.^9,38–40^ Therefore, we conclude that we further need to explore and evaluate ways to facilitate the process of personal recovery.

We did not find moderating effects of the patients’ DOI on changes in any outcome domain of personal recovery or S-QOL. This suggests that patients with SSD show stable patterns of change in personal recovery regardless of their phase of illness or history of mental health care consumption. Therefore, we could not state if there is an optimal window of opportunity of improvement in personal recovery in early psychosis or later in the course of illness. This is not in line with our meta-analysis about changes in symptoms, social functioning, and cognition (De Winter et al., manuscript submitted)^6,7^ in which we found more favorable patterns of improvement in patients with a short DOI.

In general, we found more favorable improvement in the overall scores of personal recovery and S-QOL than in specific domains of personal recovery. We found the same trend in a previous meta-analysis, where larger improvements over time were found in overall social functioning compared to more modest improvements in specific outcome domains of social functioning.^6^ A possible explanation for these findings is that global measures give a more robust, less contextual depended indication of change as it grasps multiple components of quality of life or personal recovery.^41^

### The Influence of Moderators of Changes in Personal Recovery and S-QOL

We found several moderators of change that influenced personal recovery or S-QOL.

First, we found stronger indications of improvement in connectedness and meaning in life in studies that also included patients with other SSD classifications compared to studies that exclusively included patients diagnosed with schizophrenia. This is in line with previous research indicating that higher level of symptoms and disability are negatively associated with improvement in personal recovery.^3,9,19,42^

We also observed that a low baseline level of outcomes is associated with better overall improvement in S-QOL and meaning in life. This might suggest that those with a lower level of S-QOL or meaning in life at the start of the study have a higher potential of improving on these outcomes at follow-up.

We also found that a younger age at baseline is associated with larger improvement in meaning in life. This contradicts previous findings of a healthy population where they found better meaning in life for people with an older age due to higher levels of social support.^43^ A possible explanation for our findings is that younger aged people also show better improvement in symptoms and functioning^6,7^ and are therefore more satisfied about the process of rebuilding their lives than older aged participants.

Finally, recently published studies showed larger improvement, albeit still modest, in meaning in life. This suggests that better results were achieved recently compared to more than 10 years ago.

### Contribution of the Meta-analysis

In this meta-analysis, we assessed long-term changes in personal recovery and S-QOL in SSD through quantitative assessments of personal recovery and S-QOL. Because of our focus on quantitative outcome scales, we do not grasp the personal narratives and unique processes of recovery,^8,11,12^ which is not suitable for quantifiable assessment. Current meta-analysis focused on *changes* in the individual perspective of a patient’s adaptation and behavior toward their own mental health problems and opportunities in life, using quantitative measures of personal recovery and quality of life. This gave some important insights that confirms previous research^2,22^ and what is widely experienced in clinical practice: improvement of personal recovery is possible, but quantitative improvement is on average modest at best. This might suggest that the shift toward a person-centered perspective that matches the goals and needs of patients^9,10^ has not yet been established in clinical practice. Building on this insight, a more idiographic approach of providing information concerning the individual processes of personal recovery is also a very important topic to further investigate and substantiate this statement. Therefore, for future research, we recommend to also focus on a mixed-method approach of both qualitative and quantitative evaluations to adequately investigate both the process of recovery and its outcomes.

The modest changes in personal recovery or S-QOL we observed cannot be explained by a “ceiling effect” (since at baseline relatively low levels of personal recovery or S-QOL were found) nor by a selective sample with less capacity to change. We did not find moderating effects of clinical trials or treatment targeting improvement of personal recovery or S-QOL on changes in outcomes. This finding can be explained by the fact that we assessed the combined change of the intervention and control condition. We chose to include both clinical trials and cohort studies in order to maximize the number of studies in the current meta-analysis. Importantly, regardless of the study design, exposure to treatment for this target group is inevitable as all of the included participants of the studies are recruited in mental healthcare settings. By controlling for treatment and study design as confounders, we tried to minimize the influence of treatment in a mental health care setting on the natural course of recovery as thoroughly as possible. This provides the most optimal way to give insights into the changes of personal recovery and S-QOL. Interventions are nowadays still mostly targeted at improving clinical and functional outcomes and often neglect to address personal recovery.^44^ In previous literature it is proposed that developing a focus on personal recovery in clinical practice will entail a fundamental shift in the values of mental health services.^45^ Although we were not able to establish insights concerning the context or focus of mental health care services in included studies, the modest improvement in personal recovery may suggest that mental health care services are not sufficiently equipped to support patients in their personal recovery process. Therefore, we propose to broaden the focus in mental healthcare from a clinical perspective toward a focus on personal goals and existential needs of an individual, not only during treatment, but also in the daily life of an individual. This may also involve creating opportunities to attain meaningful goals by facilitating the right conditions outside of mental health care. This could be achieved in close collaboration with organizations in the social domain and with the personal network of the patient. However, as these insights fall beyond the scope of our research, we recommend to investigate this topic in future research.

### Limitations

This meta-analysis had several limitations. First, our meta-analyses were based on a limited number of studies, especially in our analyses of overall personal recovery, hope and optimism, identity, and empowerment. This increases the chance of finding false negative results.^30^ We recommend to replicate this approach in the future, when more studies about changes in personal recovery are available, to substantiate our findings. We minimized these risks by executing Benjamini-Hochberg corrections of multiple testing.^34^ Furthermore, our included studies were executed in different settings and contexts in our meta-analysis, causing clinical heterogeneity. We controlled for most of these different contexts and settings by executing meta-regression analyses on potential moderators of outcomes (see Quality assessment and supplementary material 3). Another factor is that it is not clear when each study started registering the DOI of its sample. This could have led to modest differences between studies. Finally, our inclusion criteria were relatively strict in order to keep our study population and study design as homogeneous as possible. Therefore, several studies, assessing the course of personal recovery and quality of life with a mixed study sample of patients with multiple diagnoses, or assessed over a follow-up period shorter than 1 year, were not included in our study.

### Conclusions

In this meta-analysis, we observed modest improvement of personal recovery and quality of life. This suggests a shift is needed toward a more personal recovery-oriented perspective in mental healthcare. Therefore, future research needs to evaluate which interventions, both inside and outside the mental healthcare setting, facilitates patients with SSD in their personal recovery process.

## Supplementary Material

Supplementary material is available at https://academic.oup.com/schizophreniabulletin/.

sbae045_suppl_Supplementary_Materials

## Data Availability

The majority of relevant data and materials are presented in the tables and supplementary materials, as well as partially available in the review protocol that is submitted in PROSPERO (CRD42022377100). All other raw data is not available online. Further questions and requests about availability of the data could be sent to the corresponding author (L.d.W.; lwinter@kcphrenos.nl).
