# Supplementary material for: Long-term Changes in Personal Recovery and Quality of Life Among Patients With Schizophrenia Spectrum Disorders and Different Durations of Illness: A Meta-analysis
Source: Schizophr Bull. 2024 Apr 13;51(1):37–53. doi: 10.1093/schbul/sbae045 (PMC11661942; doi:10.1093/schbul/sbae045)
Supplement: sbae045_suppl_Supplementary_Materials [file sbae045_suppl_supplementary_materials.docx]

**Supplementary material 1.** Search history

**PsycInfo**

| **#** | **Query / limiters** |
| --- | --- |
| 1 | (Schizophrenia or Disorganized or Paranoid or Acute Schizophreniform disorder or Psychosis Schizoaffective disorder or Schizophrenia spectrum disorder Psychotic disorder).af. |
| 2 | (Delusion or Thought disturbances or Paranoia or Hallucinations or Visual or Auditory).af. |
| 3 | (Course or Prognosis or Disease or Evaluation or Rehabilitation or Remission or Recovery or Changes or Improvement or Deterioration or Development or Enhancement or Decrease or Decay or Depravation).af. |
| 4 | (Functioning or Social or Vocational or Work or Education or Relationships or Functional or Society or Symptom or Symptoms or Positive or Negative or Disorganized or Disorganization or Depression or Mood or Psychotic or Quality of life or QOL or Subjective or Well-being or Self-esteeem or Stigma or Personal or Recovery or Personal recovery or Cognition or Intelligence or IQ or Memory or Working or Long-term or Executive or Language or Motor or Perception or Processing speed or Recognition or Visuospatial).af. |
| 5 | 1 and 2 |
| 6 | 3 and 4 and 5 |
| 7 | limit 6 to (english language and abstracts and (2100 general psychology or 2224 clinical psychological testing or 2225 neuropsychological assessment or 2820 cognitive & perceptual development or 2840 psychosocial & personality development or 3000 social psychology or 3040 social perception & cognition or 3210 psychological disorders or 3213 schizophrenia & psychotic states or 3300 health & mental health treatment & prevention or 3310 psychotherapy & psychotherapeutic counseling or 3380 rehabilitation or 3384 occupational & vocational rehabilitation) and adulthood <18+ years> and ("300 adulthood <age 18 yrs and older>" or 320 young adulthood <age 18 to 29 yrs> or 340 thirties <age 30 to 39 yrs> or 360 middle age <age 40 to 64 yrs> or "380 aged <age 65 yrs and older>") and ("0100 journal" or "0110 peer-reviewed journal") and journal article and human") |
| **Results** | **5267** |

**Pubmed**

| Search number | Query | Results |
| --- | --- | --- |
| 1 | (((((((schizophrenia[MeSH Terms]) OR (disorganized schizophrenia[MeSH Terms])) OR (catatonic schizophrenia[MeSH Terms])) OR (disorders, schizophreniform[MeSH Terms])) OR (disorders, schizophrenic[MeSH Terms])) OR (disorders, schizoaffective[MeSH Terms])) OR (psychosis[MeSH Terms])) OR (disorder, psychotic[MeSH Terms]) | 152,257 |
| 2 | ((((delusion[MeSH Terms]) OR (thought disturbance[MeSH Terms])) OR (behavior, paranoid[MeSH Terms])) OR (auditory hallucination[MeSH Terms])) OR (visual hallucinations[MeSH Terms]) | 12,750 |
| 3 | (((((((((((((((course, short term[MeSH Terms]) OR (course[MeSH Terms])) OR (prognosis[MeSH Terms])) OR (evaluation[MeSH Terms])) OR (care, self rehabilitation[MeSH Terms])) OR (rehabilitation[MeSH Terms])) OR (remission[MeSH Terms])) OR (recovery[MeSH Terms])) OR (changes[MeSH Terms])) OR (improvement[MeSH Terms])) OR (deterioration[MeSH Terms])) OR (development[MeSH Terms])) OR (enhancement[MeSH Terms])) OR (decrease[MeSH Terms])) OR (decay[MeSH Terms])) OR (depravation[MeSH Terms]) | 550,904 |
| 4 | ((((((((((((((((((((((((((((((((((((((functioning[MeSH Terms]) OR (social[MeSH Terms])) OR (vocational[MeSH Terms])) OR (work[MeSH Terms])) OR (education[MeSH Terms])) OR (relationship[MeSH Terms])) OR (functional[MeSH Terms])) OR (society[MeSH Terms])) OR (friends society[MeSH Terms])) OR (symptoms[MeSH Terms])) OR (affective symptoms[MeSH Terms])) OR (positive symptoms[MeSH Terms])) OR (negative symptoms[MeSH Terms])) OR (disorganization[MeSH Terms])) OR (depression[MeSH Terms])) OR (disorder, mood[MeSH Terms])) OR (psychotic[MeSH Terms])) OR (quality of life[MeSH Terms])) OR (qol[MeSH Terms])) OR (subjective[MeSH Terms])) OR (wellbeing[MeSH Terms])) OR (self-esteem[MeSH Terms])) OR (social stigma[MeSH Terms])) OR (internalized stigma[MeSH Terms])) OR (self-stigma[MeSH Terms])) OR (personal recovery[MeSH Terms])) OR (cognition[MeSH Terms])) OR (intelligence[MeSH Terms])) OR (IQ[MeSH Terms])) OR (memory[MeSH Terms])) OR (working memory[MeSH Terms])) OR (long-term memory[MeSH Terms])) OR (executive functions[MeSH Terms])) OR (language[MeSH Terms])) OR (activity, motor[MeSH Terms])) OR (perception[MeSH Terms])) OR (processing speed[MeSH Terms])) OR (recognition[MeSH Terms])) OR (visuospatial[MeSH Terms]) | 1,655,594 |
| 5 | #1 AND #2 AND #3 AND #4 | 2862 |

**CINAHL**

| **#** | **Query** | **Limiters/Expanders** | **Results** |
| --- | --- | --- | --- |
| S1 | TI schizophrenia OR TI disorganized OR TI paranoid OR TI acute OR TI schizophreniform disorder OR TI schizoaffective disorder OR TI psychosis OR TI psychotic disorder OR TI schizophrenia spectrum OR TI delusion OR TI hallucination OR TI thought disturbance | Limiters - Abstract Available; English Language; Peer Reviewed; Research Article; Human; Journal Subset: Peer Reviewed; Publication Type: Journal Article; Age Groups: Adult: 19-44 years, Middle Aged: 45-64 years | 49,264 |
| S2 | TI course OR TI prognosis OR TI evaluation OR TI rehabilitation OR TI remission OR TI recovery OR TI changes OR TI improvement OR TI enhancement OR TI development OR TI decrease OR TI deterioration | Limiters - Abstract Available; English Language; Peer Reviewed; Research Article; Journal Subset: Peer Reviewed; Publication Type: Journal Article; Age Groups: Adult: 19-44 years, Middle Aged: 45-64 years | 13,042 |
| S3 | TI quality of life OR TI qol OR TI subjective OR TI well-being OR TI self-esteem OR TI self-efficacy OR TI empowerment OR TI stigma OR TI self-stigma OR TI personal recovery OR TI recovery | Limiters - Abstract Available; English Language; Peer Reviewed; Research Article; Journal Subset: Peer Reviewed; Publication Type: Journal Article; Age Groups: Adult: 19-44 years, Middle Aged: 45-64 years | 2,236 |
| S4 | S1 OR S2 OR S3 |  | 11,490 |
| S8 | S1 AND S2 AND S4 |  | 1568 |

**Cochrane**

| **ID** | **Search** | **Hits** |
| --- | --- | --- |
| #1 | MeSH descriptor: [Schizophrenia] explode all trees | 7876 |
| #2 | MeSH descriptor: [Schizophrenia Spectrum and Other Psychotic Disorders] explode all trees | 9619 |
| #3 | MeSH descriptor: [Psychotic Disorders] explode all trees | 3174 |
| #4 | MeSH descriptor: [Delusions] explode all trees | 175 |
| #5 | MeSH descriptor: [Hallucinations] explode all trees | 365 |
| #6 | #1 OR #2 OR #3 OR #4 OR #5 | 9795 |
| #7 | MeSH descriptor: [Disease Progression] explode all trees | 7781 |
| #8 | MeSH descriptor: [Mental Health Recovery] explode all trees | 8 |
| #9 | (course of illness):ti,ab,kw | 3268 |
| #10 | (prognosis of illness):ti,ab,kw | 1772 |
| #11 | (changes in illness):ti,ab,kw | 6552 |
| #12 | #7 OR #8 OR #9 OR #10 OR #11 | 18104 |
| #13 | (psychotic symptoms):ti,ab,kw | 3700 |
| #14 | (negative symptoms):ti,ab,kw | 13981 |
| #15 | (functioning):ti,ab,kw | 27292 |
| #16 | (social adjustment):ti,ab,kw | 2751 |
| #17 | (vocational functioning):ti,ab,kw | 293 |
| #18 | (personal recovery):ti,ab,kw | 895 |
| #19 | MeSH descriptor: [Quality of Life] this term only | 27155 |
| #20 | (self-stigma):ti,ab,kw | 167 |
| #21 | (empowerment):ti,ab,kw | 2464 |
| #22 | (self-esteem):ti,ab,kw | 3690 |
| #23 | (well-being):ti,ab,kw | 15275 |
| #24 | MeSH descriptor: [Mental Processes] explode all trees | 46642 |
| #25 | #13 OR #14 OR #15 OR #16 OR #17 OR #18 OR #19 OR #20 OR #21 OR #22 OR #23 OR #24 | 124937 |
| #26 | #6 AND #12 AND #25 | 1357 |

**Supplementary material 2.** Definitions of personal recovery and quality of life outcomes

| **1. Connectedness** | | |
| --- | --- | --- |
| *Definition:* The level of connectedness that an individual experiences with their social network. This includes experiences of peer support, relationships, being part of a community or society. | | |
| **Outcome name** | **Measurement** | **Explanation of outcome** |
| Satisfaction with family | Modified Lehman Quality of Life Inventory (QOLI-M) | QOLI-M Subjective Family Quality of Life Scale |
|  | SCAP-HQ | SCAP-HQ feelings about relationships with family members score |
| Satisfaction with relationships | Lehman's quality of life scale | Lehman's QOL satisfaction with family relations subscale |
|  |  | Lehman's QOL satisfaction with social relations subscale |
|  |  | Lehman's S-QOL family contacts subscale score |
|  |  | Lehman's S-QOL activities and social contacts subscale score |
|  | Q-LES-Q | Q-LES-Q social relationships scale |
|  | SF-36 | SCAP-HQ social relationships |
|  |  | SF-36 Social Functioning item score |
|  | WHO-QOL BREF | WHO-QOL BREF satisfaction with social relationships domain score |
|  |  | WHO-QOL BREF social relationships subscale score |
| Social cohesion | Recovery Assessment Scale (RAS) | RAS Willingness to ask for help item score |
|  |  | RAS reliance on others subscale score |
|  | Resilience Scale for Adults | RSA social competence score |
|  |  | RSA family cohesion score |
| Social integration | SWN-20 | SWN-20 social integration score |
| Social support | Social Support Questionnaire-6 | SSQ-6 total score |
| Subjective value of interpersonal relations | Heinrichs Quality of Life Scale | Heinrich's QOL interpersonal relations score |
| **2. Hope and optimism about the future** | | |
| *Definition:* The level of hope, motivation and optimism that an individual has towards its future or recovery process. This includes outcomes related to a belief in possibility of recovery, motivation to change, positive thinking and having dreams and aspirations | | |
| **Outcome name** | **Measurement** | **Explanation of outcome** |
| Hope | Integrative Hope Scale (IHS) | IHS total score |
|  | Recovery Assessment Scale | RAS hope subscale score |
|  |  | RAS Personal confidence and hope scale |
| Expectations | OPS quality of life score | OPS level of acceptability of the patients’ position in life relative to their expectations |
| Optimism | Adult Trait Hope Scale | ATHS optimism subscale score |
|  | OPS quality of life score | OPS expectation subscale |
|  | Resilience Scale for Adults | Resilience Scale for Adults (RSA) planned future subscale score |
| Sense of purpose | Heinrichs Quality of Life Scale | Heinrich's QLS Intrapsychic Foundation Subscale degree of motivation, sense of purpose, and curiosity item scores |
| **3. Identity** | | |
| *Definition:* The identity that an individual experiences towards themselves. This includes concepts such as different dimensions of identity, redefining a positive sense of identity, and overcoming (self-)stigma. | | |
| **Outcome name** | **Measurement** | **Explanation of outcome** |
| Perceived discrimination | Perceived Devaluation and Discrimination Questionnaire | PDDQ total score |
| Self-concept | Brief Core Schema Scale (BCSS) | BCSS negative self subscale score |
|  |  | BCSS negative others subscale score |
|  |  | BCSS positive self subscale score |
|  |  | BCSS positive others subscale score |
| Stigma | Burden due to Stigma Experiences | B-STE total score |
|  | ISMI | ISMI mean item score excluding stigma resistence |
| **4. Meaning in life** | | |
| *Definition:* This outcome assesses all ratings of individuals about the meaningfulness of their lives. This includes the meaning of mental health experiences, spirituality, quality of life, social roles and goals and rebuilding of their lives. | | |
| **Outcome name** | **Measurement** | **Explanation of outcome** |
| Meaning in life | Meaning in Life Questionnaire | MLQ total score |
|  | OPS | OPS position in life subscale score |
| Satisfaction with life domains | Lehman's quality of life scale | Lehman's QOLI satisfaction with leisure subscale |
|  | Q-LES-Q-18 | Q-LES-Q-18 total score |
|  | Sense of Coherence (SOC) scale | SOC total |
|  |  | SOC meaningfulness |
|  | WHO-QOL 26/BREF | WHO-QOL BREF global life satisfaction score |
|  |  | WHO-QOL 26/BREF psychological domain score |
| Subjective rating of mental health | EQ-5D | EQ-5D total score |
|  |  | EQ-5D Health Thermometer |
|  |  | EQ-5D VAS score |
|  | Hamilton Program for Schizophrenia Voices Questionnaire (HPSVQ) | HPSVQ item 7 score: how bad do they make you feel? |
| **5. Empowerment** | | |
| *Definition:* The level of empowerment and control over their own lives that individuals experience. This includes concepts such as personal responsibility, control over life and focusing upon strengths. | | |
| **Outcome name** | **Measurement** | **Explanation of outcome** |
| Control over life | Recovery Assessment Scale (RAS) | RAS No domination by symptoms subscale |
| Focus on strength and goal orientation | the Client's Assessment of Strengths, Interests, and Goals (CASIG) | CASIG total score |
|  | Recovery Assessment Scale (RAS) | RAS Goal and success orientation |
| Resilience | Connor-Davidson resilience scale (CDRISC) | CDRISC score |
|  | Resilience Scale for Adults (RSA) | RSA perception of self score |
| Sense of agency | Adult Trait Hope Scale | ATHS sense of agency subscale score |
| Self-control | SWN-20 | SWN-20 self-control subscale score |
| **6. Overall personal recovery** | | |
| *Definition:* In this outcome category we include all outcomes that comprise multiple domains of personal recovery from the CHIME framework or total scores of assessment instruments focused on overall personal recovery. | | |
| **Outcome name** | **Measurement** | **Explanation of outcome** |
| Assessment of recovery | self-report assessment of recovery | Combination of MARS and MHSIP assessment of recovery scores |
| QPR | Questionnaire About the Process of Recovery (QPR) | QPR Total score |
| RAS | Recovery Assessment Scale (RAS) | RAS total score |
| **7. Overall subjective quality of life** | | |
| *Definition:* In this outcome category we include all outcomes that comprise multiple domains of subjective quality of life or total scores of assessment instruments focused on overall subjective quality of life. | | |
| **Outcome name** | **Measurement** | **Explanation of outcome** |
| Heinrichs QLS | Heinrichs quality of life scale | Heinrichs QLS Intrapsychic Foundations |
|  |  | Heinrichs QLS total score |
| Lehman’s QLS | Lehman's quality of life scale | Lehman's quality of life scale general life satisfaction subscale |
|  |  | Lehmans QLS total score |
| LQLP | Lancashire Quality of Life profile | LQLP total score |
| MANSA | MANSA | MANSA total score |
| QOLI-M | Modified Lehman Quality of Life Inventory (QOLI-M) | QOLI-M Overall Subjective Quality of Life Scale |
|  |  | QOLI-M total score |
| SLDS | SLDS | SLDS total score |
| SWN-20 | SWN-20 | SWN-20 total score |
| WHO-QOL 26 | WHO-QOL 26 | WHO-QOL 26 total score |

**Supplementary material 3.** Meta-regression outcomes

| ***Overall personal recovery*** | | | | |
| --- | --- | --- | --- | --- |
| **Moderator (operationalization)** | **N studies** | **B** | **SE** | **p** |
| Age at baseline (higher scores representing older age at baseline) | 7 | -0.04 | 0.02 | 0.06 |
| Age at onset (higher scores representing older age at onset) | 5 | 0.00 | 0.03 | 0.96 |
| Baseline level of outcome domain: percentile score of norm group with psychotic disorder (higher scores represent better baseline level of functioning) | 7 | -0.02 | 0.01 | 0.08 |
| Baseline duration of illness | 7 | -0.32 | 0.28 | 0.29 |
| Duration of follow-up in years | 7 | -0.28 | 0.51 | 0.61 |
| Duration of Untreated Psychosis (DUP) in months: higher score representing longer DUP | 5 | -0.01 | 0.01 | 0.34 |
| Gender: % female (higher scores representing higher percentage) | 7 | 0.01 | 0.02 | 0.58 |
| Negative symptoms at baseline: percentile score of norm group with psychotic disorder (higher scores representing higher symptom severity) | 5 | -0.02 | 0.01 | 0.06 |
| Positive symptoms at baseline: percentile score of norm group with psychotic disorder (higher scores representing higher symptom severity) | 5 | -0.01 | 0.01 | 0.34 |
| Publication year (higher scores representing more recent publications) | 7 | -0.12 | 0.05 | 0.07 |
| Schizophrenia diagnosis: all participants diagnosed with schizophrenia (yes vs. no) | 5 | -0.78 | 0.75 | 0.36 |
| Treatment focused on outcome: In case there is a specific intervention used (other than antipsychotics): is the intervention specifically focused on improvement of the given outcome domain (yes/no) | 6 | -0.56 | 0.51 | 0.32 |
| ***Overall subjective quality of life*** | | | | |
| **Moderator (operationalization)** | **N studies** | **B** | **SE** | **p** |
| Age at baseline (higher scores representing older age at baseline) | 26 | -0.01 | 0.01 | 0.50 |
| Age at onset (higher scores representing older age at onset) | 21 | -0.00 | 0.02 | 0.95 |
| Antipsychotic use by all participants (yes vs. no) | 18 | 0.15 | 0.18 | 0.42 |
| Baseline duration of illness | 26 | -0.05 | 0.15 | 0.73 |
| **Baseline level of outcome domain: percentile score of norm group with psychotic disorder (higher scores represent better baseline level of functioning)** | 26 | -0.01 | 0.00 | 0.00 |
| Depressive symptoms at baseline: percentile score of norm group with psychotic disorder (higher scores representing higher symptom severity) | 7 | 0.01 | 0.00 | 0.10 |
| Duration of follow-up in years | 26 | -0.02 | 0.02 | 0.28 |
| Duration of Untreated Psychosis (DUP) in months: higher score representing longer DUP | 7 | 0.01 | 0.01 | 0.48 |
| Education level or duration: Percentage of patients with a specific education level, operationalized through ISCED criteria or the number of years of education. For both types of moderators we calculate the median and assess studies with participants with a ‘high level of education’ (level or duration is above median) or ‘low level of education’ (level or duration is below median) | 13 | 0.10 | 0.28 | 0.73 |
| Ethnicity: % Caucasian or from the country of origin (higher scores representing higher percentage) | 9 | 0.00 | 0.00 | 0.86 |
| Gender: % female (higher scores representing higher percentage) | 26 | 0.01 | 0.01 | 0.25 |
| General functioning at baseline: percentile score of norm group with psychotic disorder (higher scores represent better baseline level of functioning) | 12 | -0.01 | 0.01 | 0.08 |
| Negative symptoms at baseline: percentile score of norm group with psychotic disorder (higher scores representing higher symptom severity) | 19 | 0.00 | 0.00 | 0.24 |
| Overall symptoms at baseline: percentile score of norm group with psychotic disorder (higher scores representing higher symptom severity) | 16 | 0.01 | 0.00 | 0.24 |
| Positive symptoms at baseline: percentile score of norm group with psychotic disorder (higher scores representing higher symptom severity) | 17 | 0.00 | 0.00 | 0.30 |
| Publication year (higher scores representing more recent publications) | 26 | 0.03 | 0.01 | 0.07 |
| Schizophrenia diagnosis: all participants diagnosed with schizophrenia (yes vs. no) | 21 | 0.16 | 0.18 | 0.38 |
| Study design (Clinical trial versus cohort study) | 26 | 0.09 | 0.17 | 0.60 |
| Treatment focused on outcome: In case there is a specific intervention used (other than antipsychotics): is the intervention specifically focused on improvement of the given outcome domain (yes/no) | 18 | -0.20 | 0.20 | 0.33 |
| Vocational functioning: Percentage (%) of participants who are competitively employed at baseline (higher scores representing more participants employed) | 8 | -0.00 | 0.01 | 0.57 |
| ***Connectedness*** | | | | |
| **Moderator (operationalization)** | **N studies** | **B** | **SE** | **p** |
| Age at baseline (higher scores representing older age at baseline) | 13 | -0.01 | 0.01 | 0.20 |
| Age at onset (higher scores representing older age at onset) | 11 | -0.02 | 0.01 | 0.12 |
| Antipsychotic use by all participants (yes vs. no) | 8 | 0.03 | 0.25 | 0.90 |
| Baseline duration of illness | 13 | 0.15 | 0.16 | 0.37 |
| Baseline level of outcome domain: percentile score of norm group with psychotic disorder (higher scores represent better baseline level of functioning) | 13 | 0.01 | 0.01 | 0.30 |
| Duration of follow-up in years | 13 | -0.03 | 0.02 | 0.19 |
| Education level or duration: Percentage of patients with a specific education level, operationalized through ISCED criteria or the number of years of education. For both types of moderators we calculate the median and assess studies with participants with a ‘high level of education’ (level or duration is above median) or ‘low level of education’ (level or duration is below median) | 6 | -0.13 | 0.18 | 0.53 |
| Ethnicity: % Caucasian or from the country of origin (higher scores representing higher percentage) | 5 | 0.00 | 0.01 | 0.93 |
| Gender: % female (higher scores representing higher percentage) | 13 | -0.01 | 0.01 | 0.35 |
| General functioning at baseline: percentile score of norm group with psychotic disorder (higher scores represent better baseline level of functioning) | 6 | 0.00 | 0.01 | 0.62 |
| Negative symptoms at baseline: percentile score of norm group with psychotic disorder (higher scores representing higher symptom severity) | 9 | 0.00 | 0.01 | 0.98 |
| Overall symptoms at baseline: percentile score of norm group with psychotic disorder (higher scores representing higher symptom severity) | 7 | 0.00 | 0.00 | 0.65 |
| Positive symptoms at baseline: percentile score of norm group with psychotic disorder (higher scores representing higher symptom severity) | 8 | 0.00 | 0.00 | 0.97 |
| Publication year (higher scores representing more recent publications) | 13 | 0.00 | 0.01 | 0.90 |
| **Schizophrenia diagnosis: all participants diagnosed with schizophrenia (yes vs. no)** | 12 | 0.37 | 0.15 | 0.03 |
| Study design (Clinical trial versus cohort study) | 13 | 0.20 | 0.18 | 0.28 |
| Treatment focused on outcome: In case there is a specific intervention used (other than antipsychotics): is the intervention specifically focused on improvement of the given outcome domain (yes/no) | 8 | -0.32 | 0.18 | 0.11 |
| Vocational functioning: Percentage (%) of participants who are competitively employed at baseline (higher scores representing more participants employed) | 5 | -0.01 | 0.00 | 0.21 |
| ***Meaning in Life*** | | | | |
| **Moderator (operationalization)** | **N studies** | **B** | **SE** | **p** |
| **Age at baseline (higher scores representing older age at baseline)** | 14 | -0.01 | 0.01 | 0.02 |
| Age at onset (higher scores representing older age at onset) | 9 | -0.03 | 0.02 | 0.13 |
| Antipsychotic use by all participants (yes vs. no) | 6 | -0.26 | 0.25 | 0.34 |
| Baseline duration of illness | 14 | -0.09 | 0.09 | 0.37 |
| **Baseline level of outcome domain: percentile score of norm group with psychotic disorder (higher scores represent better baseline level of functioning)** | 14 | -0.01 | 0.00 | 0.04 |
| Duration of follow-up in years | 14 | -0.02 | 0.02 | 0.18 |
| Duration of illness subgroup overlap: The range of the duration of illness of the study sample overlaps with other duration of illness subgroups (yes vs no) | 8 | -0.13 | 0.24 | 0.61 |
| Duration of Untreated Psychosis (DUP) in months: higher score representing longer DUP | 5 | 0.01 | 0.01 | 0.27 |
| Education level or duration: Percentage of patients with a specific education level, operationalized through ISCED criteria or the number of years of education. For both types of moderators we calculate the median and assess studies with participants with a ‘high level of education’ (level or duration is above median) or ‘low level of education’ (level or duration is below median) | 8 | 0.18 | 0.19 | 0.37 |
| Gender: % female (higher scores representing higher percentage) | 14 | -0.00 | 0.01 | 0.88 |
| General functioning at baseline: percentile score of norm group with psychotic disorder (higher scores represent better baseline level of functioning) | 9 | -0.01 | 0.01 | 0.25 |
| Negative symptoms at baseline: percentile score of norm group with psychotic disorder (higher scores representing higher symptom severity) | 10 | 0.00 | 0.00 | 0.21 |
| Overall symptoms at baseline: percentile score of norm group with psychotic disorder (higher scores representing higher symptom severity) | 8 | 0.01 | 0.00 | 0.07 |
| Positive symptoms at baseline: percentile score of norm group with psychotic disorder (higher scores representing higher symptom severity) | 9 | 0.01 | 0.00 | 0.07 |
| **Publication year (higher scores representing more recent publications)** | 14 | 0.02 | 0.01 | 0.02 |
| **Schizophrenia diagnosis: all participants diagnosed with schizophrenia (yes vs. no)** | 10 | 0.40 | 0.12 | 0.01 |
| Study design (Clinical trial versus cohort study) | 14 | 0.04 | 0.14 | 0.77 |
| Treatment focused on outcome: In case there is a specific intervention used (other than antipsychotics): is the intervention specifically focused on improvement of the given outcome domain (yes/no) | 6 | -0.01 | 0.20 | 0.98 |

**Supplementary material 4.** QUIPS quality assessment of all included studies

| **Study name** | **Study participation** | **Study attrition** | **Prognostic factor measurement** | **Outcome measurement** | **Study confounding** | **Statistical analysis and report** |
| --- | --- | --- | --- | --- | --- | --- |
| Addington 2000 | Moderate | Low | Low | Low | High | High |
| Beaudoin 2022 | Low | Moderate | Low | Low | High | Low |
| Buonocore 2018 | Moderate | Moderate | Low | Low | High | Low |
| Chan 2003 | Low | Moderate | Unclear | Low | High | Moderate |
| Chien 2014 | Low | Low | Low | Low | High | Low |
| Conley 2007 | Low | Low | Low | Low | Moderate | Low |
| Dellazizzo 2021 | Moderate | Low | Low | Low | High | Low |
| Fernández-Modamio 2021 | Moderate | High | Low | Low | Moderate | Moderate |
| Fowler 2012 | Moderate | Moderate | Low | Low | High | Moderate |
| Fowler 2018 | Moderate | Moderate | Unclear | Low | High | Low |
| Galderisi 2020 | Low | Low | Moderate | Low | High | Low |
| Godin 2019 | Low | Moderate | Low | Low | Moderate | Low |
| Gorna 2008 | Moderate | Moderate | Low | Low | Low | Low |
| Gumley 2022 | Low | Moderate | Low | Moderate | High | Low |
| Hayhurst 2014 | Moderate | Moderate | Low | Low | Low | Low |
| Heering 2015 | Moderate | Moderate | Moderate | Low | High | Low |
| Ito 2015 | Low | Low | Moderate | Low | Low | Low |
| Jørgensen 2015 | Low | Low | Unclear | Low | Low | Low |
| Kane 2016 | Low | High | Low | Moderate | Moderate | Low |
| Kelly 2009 | Moderate | High | Unclear | Low | Low | Low |
| Kim 2019 | Moderate | Low | Unclear | Moderate | Low | Low |
| Kumazaki 2012 | Moderate | Moderate | Moderate | Low | Low | Low |
| Lasser 2005 | Moderate | Moderate | Moderate | Low | Low | Low |
| Lee 2023 | Moderate | Moderate | Low | Low | Moderate | Low |
| Litman 2023 | Low | Low | Low | Low | Low | Low |
| Liu 2023 | Moderate | Moderate | Moderate | Low | Moderate | Moderate |
| Lopez-Morinigo 2022 | Moderate | Low | Moderate | Low | Moderate | Moderate |
| Marino 2015 | Low | High | Moderate | Low | Moderate | Moderate |
| McNeely 2022 | Moderate | Low | Low | Low | Moderate | Moderate |
| Moncrieff 2023 | Low | Moderate | Low | Low | Low | Low |
| Morrison 2018 | Low | Low | Unclear | Low | Low | Low |
| Na 2016 | Low | Low | Unclear | Low | High | Low |
| Neill 2022 | Moderate | High | Moderate | Low | Moderate | Low |
| Ortega 2020 | Moderate | Moderate | Low | Low | Moderate | Moderate |
| Prouteau 2005 | Low | Low | Low | Low | Low | Low |
| Rowland 2018 | Low | Moderate | Low | Low | Low | Low |
| Salyers 2014 | Low | High | Unclear | Low | Low | Low |
| Schmidt 2017 | Low | Low | Low | Low | Moderate | Low |
| Sikira 2021 | Low | Low | Low | Low | High | Moderate |
| Tabo 2017 | Moderate | High | Moderate | Moderate | High | Low |
| Usui 2022 | Low | Low | Moderate | Low | Low | Low |
| Veerman 2016 | Low | Low | Low | Low | Low | Moderate |
| Wilson-d'Almeida 2013 | Low | Low | Low | Low | Low | Low |
| Wunderink 2009 | Low | Moderate | Low | Low | Low | Low |
| Xie 2005 | Low | Low | Unclear | Low | High | Low |
| Zäske 2018 | Moderate | High | Low | Low | Moderate | Low |

**Supplementary material 5.** Sensitivity analysis QUIPS outcomes

| **Connectedness** | | | | | | |
| --- | --- | --- | --- | --- | --- | --- |
| QUIPS outcome | Rating | K (studies (outcomes)) | N (baseline-FU) | Effect size (95% CI)* and magnitude of effect** | K (%) large effect**  [+/-]*** | Heterogeneity  (I^2^ (95%CI))* |
| Study participation | Low | 10 | 4274 - 2637 | *d* = **0.14** [N] (0.03 – 0.25) | + = 0 / - = 0 | **I^2^ = 91%** (86-94%) |
|  | Moderate | 4 | 1011 - 880 | *d* = 0.11 [N] (-0.52 – 0.74) | + = 1 / - = 0 | **I^2^ = 98%** (96-99%) |
|  | **Subgroup differences between ratings** | | | *χ^2^* = 0.01; *df* = 1; *p* = 0.91 | | |
| Study attrition | Low | 7 | 3782 - 2370 | *d* = 0.08 [N] (-0.01 – 0.17) | + = 0 / - = 0 | **I^2^ = 86%** (75-93%) |
|  | Moderate | 4 | 737 - 717 | *d* = 0.18 [N] (-0.38 – 0.74) | + = 1 / - = 0 | **I^2^ = 95%** (90-98%) |
|  | High | 3 | 766 - 430 | *d* = 0.26 [S] (-0.31 – 0.82) | + = 0 / - = 0 | I^2^ = 97% (94-99%) |
|  | **Subgroup differences between ratings** | | | *χ^2^* = 0.47; *df* = 2; *p* = 0.79 | | |
| Prognostic factor measurement | Low | 6 | 3326 - 1937 | *d* = 0.06 [N] (-0.16 – 0.28) | + = 0 / - = 0 | **I^2^ = 95%** (92-97%) |
|  | Moderate | 5 | 1681 - 1310 | *d* = 0.35 [S] (-0.13 – 0.83) | + = 1 / - = 0 | **I^2^ = 97%** (95-98%) |
|  | Unclear | 3 | 278 - 270 | *d* = 0.07 [N] (-0.01 – 0.15) | + = 0 / - = 0 | I^2^ = 38% (0-77%) |
|  | **Subgroup differences between ratings** | | | *χ^2^* = 1.32; *df* = 2; *p* = 0.52 | | |
| Outcome measurement | Low | 13 | 4881 - 3290 | *d* = 0.12 [N] (-0.01 – 0.26)^M^ | + = 1 / - = 0 | **I^2^ = 95%** (92-96%) |
|  | Moderate | 1 | 404 - 227 | *d* = **0.46** [S] (0.36 – 0.56)^L^ | + = 0 / - = 0 | Not Applicable |
|  | **Subgroup differences between ratings** | | | *χ^2^* = 15.20; *df* = 1; *p* < 0.01 | | |
| Study confounding | Low | 6 | 1087 - 1019 | *d* = 0.28 [S] (-0.00 – 0.57) | + = 1 / - = 0 | **I^2^ = 94%** (90-97%) |
|  | Moderate | 4 | 2994 - 1597 | *d* = 0.16 [N] (-0.16 – 0.48) | + = 0 / - = 0 | **I^2^ = 96%** (93-98%) |
|  | High | 4 | 1204 - 901 | *d* = 0.07 [N] (-0.08 – 0.07) | + = 0 / - = 0 | I^2^ = 59% (0-84%) |
|  | **Subgroup differences between ratings** | | | *χ^2^* = 4.32; *df* = 2; *p* = 0.12 | | |
| Statistical analysis and report | Low | 2 | 274 - 234 | *d* = **0.35** [S] (0.24 – 0.46)^H^ | + = 0 / - = 0 | I^2^ = 0% (NA) |
|  | Moderate | 3 | 387 - 228 | *d* = 0.06 [N] (-0.34 – 0.45) | + = 0 / - = 0 | **I^2^ = 80%** (22-95%) |
|  | High | 9 | 4624 - 3055 | *d* = 0.14 [N] (-0.02 – 0.30)^L^ | + = 1 / - = 0 | **I^2^ = 96%** (95-98%) |
|  | **Subgroup differences between ratings** | | | *χ^2^* = 5.82; *df* = 2; *p* = 0.05 | | |

**Abbreviations:** CI = Confidence Interval; FU = Follow-up; L = Large effect; M = Medium effect; N = No effect; S = Small effect

**Note:**

***** Outcomes in bold are significant (p < 0.05);

** N = No effect (SMD >-0.20 - <0.20; OR >0.67 - <1.5); S = Small effect (SMD ≤-0.20 and >-0.50 - ≥0.20 and <0.50; OR ≤0.67 and >0.29 - ≤1.5 and <3.5); M = Medium effect (SMD ≤-0.50 and >-0.80 - ≥0.50 and <0.80; OR ≤0.29 and >0.20 - ≤3.5 and <5) ; L = Large effect (SMD <-0.80 - >0.80; OR <0.20 - >5)

*** + = improvement of outcome at follow-up; - = deterioration of outcome at follow-up

**** L = significant different from low ROB; M = significant different from moderate ROB; H = significant different from high ROB

| **Hope and optimism about the future** | | | | | | |
| --- | --- | --- | --- | --- | --- | --- |
| QUIPS outcome | Rating | K (studies (outcomes)) | N (baseline-FU) | Effect size (95% CI)* and magnitude of effect** | K (%) large effect**  [+/-]*** | Heterogeneity  (I^2^ (95%CI))* |
| Study participation | Low | 5 | 1848 - 1491 | *d* = **0.08** [N] (0.02 – 0.14) | + = 0 / - = 0 | I^2^ = 43% (0-69%) |
|  | Moderate | 2 | 186 - 133 | *d* = -0.06 [N] (-0.29 – 0.16) | + = 0 / - = 0 | I^2^ = 43% (NA) |
|  | **Subgroup differences between ratings** | | | *χ^2^* = 1.44; *df* = 1; *p* = 0.23 | | |
| Study attrition | Low | 4 | 1682 - 1368 | *d* = 0.06 [N] (-0.01 – 0.13) | + = 0 / - = 0 | I^2^ = 39% (0-70%) |
|  | Moderate | 1 | 135 - 93 | *d* = -0.11 [N] (-0.37 – 0.15) | + = 0 / - = 0 | Not Applicable |
|  | High | 2 | 217 - 163 | *d* = **0.13** [N] (0.00 – 0.26) | + = 0 / - = 0 | I^2^ = 22% (NA) |
|  | **Subgroup differences between ratings** | | | *χ^2^* = 2.65; *df* = 2; *p* = 0.27 | | |
| Prognostic factor measurement | Low | 3 | 458 - 439 | *d* = 0.08 [N] (-0.03 – 0.19) | + = 0 / - = 0 | I^2^ = 36% (0-75%) |
|  | Moderate | 1 | 404 - 404 | *d* = **0.19** [N] (0.06 – 0.32)^H^ | + = 0 / - = 0 | Not Applicable |
|  | Unclear | 3 | 1172 - 781 | *d* = 0.01 [N] (-0.08 – 0.10)^M^ | + = 0 / - = 0 | I^2^ = 0% (0-93%) |
|  | **Subgroup differences between ratings** | | | *χ^2^* = 4.88; *df* = 2; *p* = 0.09 | | |
| Outcome measurement | Low | 6 | 1933 - 1531 | *d* = 0.05 [N] (-0.02 – 0.11) | + = 0 / - = 0 | I^2^ = 23% (0-43%) |
|  | Moderate | 1 | 101 - 93 | *d* = **0.19** [N] (0.03 – 0.35) | + = 0 / - = 0 | Not Applicable |
|  | **Subgroup differences between ratings** | | | *χ^2^* = 2.43; *df* = 1; *p* = 0.12 | | |
| Study confounding | Low | 3 | 1343 - 994 | *d* = 0.01 [N] (-0.06 – 0.09) | + = 0 / - = 0 | I^2^ = 0% (0-63%) |
|  | Moderate | 2 | 152 - 133 | *d* = **0.17** [N] (0.02 – 0.32) | + = 0 / - = 0 | I^2^ = 0% (NA) |
|  | High | 2 | 539 - 497 | *d* = **0.13** [N] (0.01 – 0.25) | + = 0 / - = 0 | I^2^ = 74% (NA) |
|  | **Subgroup differences between ratings** | | | *χ^2^* = 4.43; *df* = 2; *p* = 0.11 | | |
| Statistical analysis and report | Low | 6 | 1983 - 1584 | *d* = **0.07** [N] (0.01 – 0.13) | + = 0 / - = 0 | I^2^ = 44% (5-67%) |
|  | Moderate | 1 | 51 - 40 | *d* = 0.05 [N] (-0.36 – 0.46) | + = 0 / - = 0 | Not Applicable |
|  | **Subgroup differences between ratings** | | | *χ^2^* = 0.01; *df* = 1; *p* = 0.93 | | |

***** Outcomes in bold are significant (p < 0.05);

** N = No effect (SMD >-0.20 - <0.20; OR >0.67 - <1.5); S = Small effect (SMD ≤-0.20 and >-0.50 - ≥0.20 and <0.50; OR ≤0.67 and >0.29 - ≤1.5 and <3.5); M = Medium effect (SMD ≤-0.50 and >-0.80 - ≥0.50 and <0.80; OR ≤0.29 and >0.20 - ≤3.5 and <5) ; L = Large effect (SMD <-0.80 - >0.80; OR <0.20 - >5)

*** + = improvement of outcome at follow-up; - = deterioration of outcome at follow-up

**** L = significant different from low ROB; M = significant different from moderate ROB; H = significant different from high ROB

| **Identity** | | | | | | |
| --- | --- | --- | --- | --- | --- | --- |
| QUIPS outcome | Rating | K (studies (outcomes)) | N (baseline-FU) | Effect size (95% CI)* and magnitude of effect** | K (%) large effect**  [+/-]*** | Heterogeneity  (I^2^ (95%CI))* |
| Study participation | Low | 2 | 946 - 643 | *d* = **0.21** [S] (0.11 – 0.31) | + = 0 / - = 0 | I^2^ = 0% (NA) |
|  | Moderate | 2 | 303 - 231 | *d* = **0.20** [S] (0.11 – 0.28) | + = 0 / - = 0 | I^2^ = 74% (NA) |
|  | **Subgroup differences between ratings** | | | *χ^2^* = 0.04; *df* = 1; *p* = 0.84 | | |
| Study attrition | Low | 2 | 946 - 643 | *d* = **0.21** [S] (0.11 – 0.31) | + = 0 / - = 0 | I^2^ = 0% (NA) |
|  | Moderate | 1 | 255 - 207 | *d* = **0.22** [S] (0.13 – 0.31) | + = 0 / - = 0 | Not Applicable |
|  | High | 1 | 48 - 24 | *d* = -0.14 [N] (-0.48 – 0.20) | + = 0 / - = 0 | Not Applicable |
|  | **Subgroup differences between ratings** | | | *χ^2^* = 3.96; *df* = 2; *p* = 0.14 | | |
| Prognostic factor measurement | Low | 2 | 303 - 231 | *d* = **0.20** [S] (0.11 – 0.28) | + = 0 / - = 0 | I^2^ = 74% (NA) |
|  | Moderate | 1 | 921 - 618 | *d* = **0.23** [S] (0.13 – 0.23) | + = 0 / - = 0 | Not Applicable |
|  | Unclear | 1 | 25 - 25 | *d* = 0.08 [N] (-0.19 – 0.35) | + = 0 / - = 0 | Not Applicable |
|  | **Subgroup differences between ratings** | | | *χ^2^* = 1.04; *df* = 2; *p* = 0.59 | | |
| Outcome measurement | Low | 4 | 1249 - 874 | *d* = **0.20** [S] (0.14 – 0.27) | + = 0 / - = 0 | I^2^ = 39% (0-70%) |
|  | **Subgroup differences between ratings** | | | Not Applicable | | |
| Study confounding | Moderate | 1 | 48 - 24 | *d* = -0.14 [N] (-0.48 – 0.20) | + = 0 / - = 0 | Not Applicable |
|  | High | 3 | 1201 - 850 | *d* = **0.22** [S] (0.15 – 0.28) | + = 0 / - = 0 | I^2^ = 0% (0-93%) |
|  | **Subgroup differences between ratings** | | | *χ^2^* = 3.94; *df* = 1; *p* = 0.05 | | |
| Statistical analysis and report | Low | 1 | 255 - 207 | *d* = **0.22** [S] (0.13 – 0.31) | + = 0 / - = 0 | Not Applicable |
|  | Moderate | 3 | 994 - 667 | *d* = **0.18** [N] (0.09 – 0.28) | + = 0 / - = 0 | I^2^ = 57% (0-99%) |
|  | **Subgroup differences between ratings** | | | *χ^2^* = 0.28; *df* = 1; *p* = 0.60 | | |

***** Outcomes in bold are significant (p < 0.05);

** N = No effect (SMD >-0.20 - <0.20; OR >0.67 - <1.5); S = Small effect (SMD ≤-0.20 and >-0.50 - ≥0.20 and <0.50; OR ≤0.67 and >0.29 - ≤1.5 and <3.5); M = Medium effect (SMD ≤-0.50 and >-0.80 - ≥0.50 and <0.80; OR ≤0.29 and >0.20 - ≤3.5 and <5) ; L = Large effect (SMD <-0.80 - >0.80; OR <0.20 - >5)

*** + = improvement of outcome at follow-up; - = deterioration of outcome at follow-up

**** L = significant different from low ROB; M = significant different from moderate ROB; H = significant different from high ROB

| **Meaning in life** | | | | | | |
| --- | --- | --- | --- | --- | --- | --- |
| QUIPS outcome | Rating | K (studies (outcomes)) | N (baseline-FU) | Effect size (95% CI)* and magnitude of effect** | K (%) large effect**  [+/-]*** | Heterogeneity  (I^2^ (95%CI))* |
| Study participation | Low | 8 | 1765 - 1257 | *d* = **0.18** [N] (0.06 – 0.31) | + = 0 / - = 0 | **I^2^ = 81%** (66-89%) |
|  | Moderate | 7 | 591 - 517 | *d* = **0.18** [N] (0.01 – 0.34) | + = 0 / - = 0 | **I^2^ = 61%** (30-78%) |
|  | **Subgroup differences between ratings** | | | *χ^2^* = 0.01; *df* = 1; *p* = 0.94 | | |
| Study attrition | Low | 6 | 775 - 764 | *d* = **0.25** [S] (0.11 – 0.40) | + = 0 / - = 0 | **I^2^ = 78%** (55-89%) |
|  | Moderate | 9 | 1581 - 1010 | *d* = 0.13 [N] (-0.01 – 0.27) | + = 0 / - = 0 | **I^2^ = 71%** (52-83%) |
|  | **Subgroup differences between ratings** | | | *χ^2^* = 1.47; *df* = 1; *p* = 0.23 | | |
| Prognostic factor measurement | Low | 8 | 1766 - 1247 | *d* = **0.19** [N] (0.07 – 0.32) | + = 0 / - = 0 | **I^2^ = 71%** (49-84%) |
|  | Moderate | 3 | 191 - 171 | *d* = 0.30 [S] (-0.18 – 0.78) | + = 0 / - = 0 | **I^2^ = 80%** (24-95%) |
|  | Unclear | 3 | 312 - 269 | *d* = 0.02 [N] (-0.24 – 0.29) | + = 0 / - = 0 | **I^2^ = 85%** (44-96%) |
|  | **Subgroup differences between ratings** | | | *χ^2^* = 1.56; *df* = 2; *p* = 0.46 | | |
| Outcome measurement | Low | 13 | 2199 - 1633 | *d* = **0.18** [N] (0.07 – 0.28) | + = 0 / - = 0 | **I^2^ = 76%** (64-84%) |
|  | Moderate | 2 | 157 - 141 | *d* = **0.21** [S] (0.01 – 0.42) | + = 0 / - = 0 | I^2^ = 49% (NA) |
|  | **Subgroup differences between ratings** | | | *χ^2^* = 0.10; *df* = 1; *p* = 0.75 | | |
| Study confounding | Low | 6 | 896 - 829 | *d* = 0.20 [S] (-0.01 – 0.41) | + = 0 / - = 0 | **I^2^ = 82%** (63-91%) |
|  | Moderate | 5 | 1078 - 622 | *d* = **0.28** [S] (0.12 – 0.45) | + = 0 / - = 0 | I^2^ = 56% (6-79%) |
|  | High | 4 | 382 - 323 | *d* = 0.05 [N] (-0.13 – 0.24) | + = 0 / - = 0 | **I^2^ = 78%** (39-92%) |
|  | **Subgroup differences between ratings** | | | *χ^2^* = 3.41; *df* = 2; *p* = 0.18 | | |
| Statistical analysis and report | Low | 11 | 2143 - 1572 | *d* = **0.18** [N] (0.08 – 0.28) | + = 0 / - = 0 | **I^2^ = 72%** (56-82%) |
|  | Moderate | 4 | 213 - 202 | *d* = 0.19 [N] (-0.13 – 0.51) | + = 0 / - = 0 | **I^2^ = 82%** (51-93%) |
|  | **Subgroup differences between ratings** | | | *χ^2^* = 0.00; *df* = 1; *p* = 0.95 | | |

***** Outcomes in bold are significant (p < 0.05);

** N = No effect (SMD >-0.20 - <0.20; OR >0.67 - <1.5); S = Small effect (SMD ≤-0.20 and >-0.50 - ≥0.20 and <0.50; OR ≤0.67 and >0.29 - ≤1.5 and <3.5); M = Medium effect (SMD ≤-0.50 and >-0.80 - ≥0.50 and <0.80; OR ≤0.29 and >0.20 - ≤3.5 and <5) ; L = Large effect (SMD <-0.80 - >0.80; OR <0.20 - >5)

*** + = improvement of outcome at follow-up; - = deterioration of outcome at follow-up

**** L = significant different from low ROB; M = significant different from moderate ROB; H = significant different from high ROB

| **Empowerment** | | | | | | |
| --- | --- | --- | --- | --- | --- | --- |
| QUIPS outcome | Rating | K (studies (outcomes)) | N (baseline-FU) | Effect size (95% CI)* and magnitude of effect** | K (%) large effect**  [+/-]*** | Heterogeneity  (I^2^ (95%CI))* |
| Study participation | Low | 5 | 1317 - 967 | *d* = -0.05 [N] (-0.11 – 0.01) | + = 0 / - = 0 | **I^2^ = 97%** (94-98%) |
|  | Moderate | 1 | 135 - 93 | *d* = **-0.29** [N] (-0.55 – -0.03) | + = 0 / - = 0 | Not Applicable |
|  | **Subgroup differences between ratings** | | | *χ^2^* = 2.99; *df* = 1; *p* = 0.08 | | |
| Study attrition | Low | 5 | 1317 - 967 | *d* = -0.05 [N] (-0.11 – 0.01) | + = 0 / - = 0 | **I^2^ = 97%** (94-98%) |
|  | Moderate | 1 | 135 - 93 | *d* = **-0.29** [N] (-0.55 – -0.03) | + = 0 / - = 0 | Not Applicable |
|  | **Subgroup differences between ratings** | | | *χ^2^* = 2.99; *df* = 1; *p* = 0.08 | | |
| Prognostic factor measurement | Low | 2 | 270 - 230 | *d* = **0.22** [S] (0.12 – 0.33)^M^ | + = 0 / - = 0 | I^2^ = 0% (NA) |
|  | Moderate | 1 | 921 - 618 | *d* = **-0.54** [M] (-0.64 – -0.44)^LU^ | + = 0 / - = 0 | Not Applicable |
|  | Unclear | 3 | 261 - 212 | *d* = 0.10 [N] (-0.00 – 0.20)^M^ | + = 0 / - = 0 | **I^2^ = 79%** (22-95%) |
|  | **Subgroup differences between ratings** | | | *χ^2^* = 119.84; *df* = 2; *p* < 0.01 | | |
| Outcome measurement | Low | 6 | 1452 - 1060 | *d* = **-0.06** [N] (-0.12 – -0.00) | + = 0 / - = 0 | **I^2^ = 96%** (94-98%) |
|  | **Subgroup differences between ratings** | | | Not Applicable | | |
| Study confounding | Low | 3 | 371 - 324 | *d* = **0.20** [S] (0.12 – 0.28) | + = 0 / - = 0 | I^2^ = 0% (0-95%) |
|  | High | 3 | 1081 - 736 | *d* = **-0.47** [S] (-0.56 – -0.37) | + = 0 / - = 0 | **I^2^ = 84%** (40-96%) |
|  | **Subgroup differences between ratings** | | | *χ^2^* = 116.83; *df* = 1; *p* < 0.01 | | |
| Statistical analysis and report | Low | 6 | 1452 - 1060 | *d* = **-0.06** [N] (-0.12 – -0.00) | + = 0 / - = 0 | **I^2^ = 96%** (94-98%) |
|  | **Subgroup differences between ratings** | | | Not Applicable | | |

***** Outcomes in bold are significant (p < 0.05);

** N = No effect (SMD >-0.20 - <0.20; OR >0.67 - <1.5); S = Small effect (SMD ≤-0.20 and >-0.50 - ≥0.20 and <0.50; OR ≤0.67 and >0.29 - ≤1.5 and <3.5); M = Medium effect (SMD ≤-0.50 and >-0.80 - ≥0.50 and <0.80; OR ≤0.29 and >0.20 - ≤3.5 and <5) ; L = Large effect (SMD <-0.80 - >0.80; OR <0.20 - >5)

*** + = improvement of outcome at follow-up; - = deterioration of outcome at follow-up

**** L = significant different from low ROB; M = significant different from moderate ROB; H = significant different from high ROB

| **Overall personal recovery** | | | | | | |
| --- | --- | --- | --- | --- | --- | --- |
| QUIPS outcome | Rating | K (studies (outcomes)) | N (baseline-FU) | Effect size (95% CI)* and magnitude of effect** | K (%) large effect**  [+/-]*** | Heterogeneity  (I^2^ (95%CI))* |
| Study participation | Low | 8 | 1200 - 810 | *d* = **0.32** [S] (0.24 – 0.40) | + = 0 / - = 0 | **I^2^ = 81%** (66-89%) |
|  | **Subgroup differences between ratings** | | | Not Applicable | | |
| Study attrition | Low | 3 | 300 - 277 | *d* = **0.43** [S] (0.30 – 0.56)^M^ | + = 0 / - = 0 | **I^2^ = 88%** (59-97%) |
|  | Moderate | 2 | 317 - 217 | *d* = 0.05 [N] (-0.14 – 0.25)^LH^ | + = 0 / - = 0 | **I^2^ = 78%** (NA) |
|  | High | 3 | 583 - 316 | *d* = **0.32** [S] (0.20 – 0.44)^M^ | + = 0 / - = 0 | **I^2^ = 66%** (0-91%) |
|  | **Subgroup differences between ratings** | | | *χ^2^* = 9.88; *df* = 2; *p* < 0.01 | | |
| Prognostic factor measurement | Low | 4 | 845 - 572 | *d* = **0.33** [S] (0.21 – 0.45) | + = 0 / - = 0 | **I^2^ = 84%** (56-94%) |
|  | Moderate | 1 | 63 - 15 | *d* = 0.36 [S] (-0.04 – 0.76) | + = 0 / - = 0 | Not Applicable |
|  | Unclear | 3 | 292 - 223 | *d* = **0.31** [S] (0.20 – 0.42) | + = 0 / - = 0 | **I^2^ = 89%** (64-97%) |
|  | **Subgroup differences between ratings** | | | *χ^2^* = 0.09; *df* = 2; *p* = 0.96 | | |
| Outcome measurement | Low | 6 | 724 - 523 | *d* = **0.33** [S] (0.23 – 0.44) | + = 0 / - = 0 | **I^2^ = 83%** (66-92%) |
|  | Moderate | 2 | 476 - 287 | *d* = **0.30** [S] (0.18 – 0.43) | + = 0 / - = 0 | **I^2^ = 86%** (NA) |
|  | **Subgroup differences between ratings** | | | *χ^2^* = 0.13; *df* = 1; *p* = 0.72 | | |
| Study confounding | Low | 4 | 537 - 384 | *d* = **0.30** [S] (0.18 – 0.41) | + = 0 / - = 0 | **I^2^ = 88%** (69-95%) |
|  | Moderate | 2 | 467 - 246 | *d* = **0.43** [S] (0.28 – 0.58) | + = 0 / - = 0 | I^2^ = 0% (NA) |
|  | High | 2 | 196 - 180 | *d* = **0.22** [S] (0.05 – 0.40) | + = 0 / - = 0 | **I^2^ = 88%** (NA) |
|  | **Subgroup differences between ratings** | | | *χ^2^* = 3.34; *df* = 2; *p* = 0.19 | | |
| Statistical analysis and report | Low | 7 | 1137 - 795 | *d* = **0.32** [S] (0.24 – 0.40) | + = 0 / - = 0 | **I^2^ = 84%** (69-91%) |
|  | Moderate | 1 | 63 - 16 | *d* = 0.36 [S] (-0.04 – 0.76) | + = 0 / - = 0 | Not Applicable |
|  | **Subgroup differences between ratings** | | | *χ^2^* = 0.04; *df* = 1; *p* = 0.84 | | |

***** Outcomes in bold are significant (p < 0.05);

** N = No effect (SMD >-0.20 - <0.20; OR >0.67 - <1.5); S = Small effect (SMD ≤-0.20 and >-0.50 - ≥0.20 and <0.50; OR ≤0.67 and >0.29 - ≤1.5 and <3.5); M = Medium effect (SMD ≤-0.50 and >-0.80 - ≥0.50 and <0.80; OR ≤0.29 and >0.20 - ≤3.5 and <5) ; L = Large effect (SMD <-0.80 - >0.80; OR <0.20 - >5)

*** + = improvement of outcome at follow-up; - = deterioration of outcome at follow-up

**** L = significant different from low ROB; M = significant different from moderate ROB; H = significant different from high ROB

| **Overall subjective quality of life** | | | | | | |
| --- | --- | --- | --- | --- | --- | --- |
| QUIPS outcome | Rating | K (studies (outcomes)) | N (baseline-FU) | Effect size (95% CI)* and magnitude of effect** | K (%) large effect**  [+/-]*** | Heterogeneity  (I^2^ (95%CI))* |
| Study participation | Low | 13 | 4692 - 3213 | *d* = **0.35** [S] (0.21 – 0.49) | + = 1 / - = 0 | **I^2^ = 92%** (88-94%) |
|  | Moderate | 14 | 2029 - 1703 | *d* = **0.33** [S] (0.12 – 0.53) | + = 2 / - = 0 | **I^2^ = 91%** (86-94%) |
|  | **Subgroup differences between ratings** | | | *χ^2^* = 0.03; *df* = 1; *p* = 0.86 | | |
| Study attrition | Low | 10 | 3000 - 1801 | *d* = **0.31** [S] (0.13 – 0.50) | + = 1 / - = 0 | **I^2^ = 93%** (89-95%) |
|  | Moderate | 9 | 2543 - 2381 | *d* = **0.17** [N] (0.04 – 0.31)^H^ | + = 0 / - = 0 | **I^2^ = 82%** (69-89%) |
|  | High | 8 | 1187 - 734 | *d* = **0.60** [M] (0.26 – 0.94)^M^ | + = 2 / - = 0 | **I^2^ = 93%** (88-96%) |
|  | **Subgroup differences between ratings** | | | *χ^2^* = 5.78; *df* = 2; *p* = 0.06 | | |
| Prognostic factor measurement | Low | 16 | 5277 - 3649 | *d* = **0.21** [S] (0.08 – 0.34) | + = 0 / - = 0 | **I^2^ = 90%** (85-93%) |
|  | Moderate | 7 | 1060 - 944 | *d* = **0.59** [M] (0.21 – 0.97) | + = 2 / - = 0 | **I^2^ = 93%** (87-96%) |
|  | Unclear | 4 | 384 - 323 | *d* = **0.48** [S] (0.23 – 0.74) | + = 1 / - = 0 | **I^2^ = 84%** (56-94%) |
|  | **Subgroup differences between ratings** | | | *χ^2^* = 6.07; *df* = 2; *p* = 0.05 | | |
| Outcome measurement | Low | 25 | 6197 - 4565 | *d* = **0.27** [S] (0.17 – 0.37) | + = 2 / - = 0 | **I^2^ = 87%** (83-90%) |
|  | Moderate | 2 | 524 - 351 | *d* = **1.09** [L] (0.33 – 1.84 | + = 1 / - = 0 | **I^2^ = 95%** (NA) |
|  | **Subgroup differences between ratings** | | | *χ^2^* = 4.41; *df* = 1; *p* < 0.05 | | |
| Study confounding | Low | 11 | 1388 - 1125 | *d* = **0.33** [S] (0.16 – 0.50) | + = 1 / - = 0 | **I^2^ = 87%** (79-91%) |
|  | Moderate | 8 | 3215 - 1747 | *d* = **0.39** [S] (0.13 – 0.65) | + = 1 / - = 0 | **I^2^ = 92%** (87-95%) |
|  | High | 8 | 2118 - 2044 | *d* = **0.31** [S] (0.10 – 0.53) | + = 1 / - = 0 | **I^2^ = 94%** (90-96%) |
|  | **Subgroup differences between ratings** | | | *χ^2^* = 0.20; *df* = 2; *p* = 0.90 | | |
| Statistical analysis and report | Low | 21 | 6155 - 4545 | *d* = **0.38** [S] (0.25 – 0.51)^H^ | + = 3 / - = 0 | **I^2^ = 93%** (90-94%) |
|  | Moderate | 5 | 486 - 306 | *d* = **0.26** [S] (0.09 – 0.44)^H^ | + = 0 / - = 0 | I^2^ = 44% (0-70%) |
|  | High | 1 | 80 - 65 | *d* = **-0.50** [M] (-0.83 – -0.17)^LM^ | + = 0 / - = 0 | Not Applicable |
|  | **Subgroup differences between ratings** | | | *χ^2^* = 23.88; *df* = 2; *p* < 0.01 | | |

***** Outcomes in bold are significant (p < 0.05);

** N = No effect (SMD >-0.20 - <0.20; OR >0.67 - <1.5); S = Small effect (SMD ≤-0.20 and >-0.50 - ≥0.20 and <0.50; OR ≤0.67 and >0.29 - ≤1.5 and <3.5); M = Medium effect (SMD ≤-0.50 and >-0.80 - ≥0.50 and <0.80; OR ≤0.29 and >0.20 - ≤3.5 and <5) ; L = Large effect (SMD <-0.80 - >0.80; OR <0.20 - >5)

*** + = improvement of outcome at follow-up; - = deterioration of outcome at follow-up

**** L = significant different from low ROB; M = significant different from moderate ROB; H = significant different from high ROB

**Supplementary material 6.** Overview of funnel plots

**
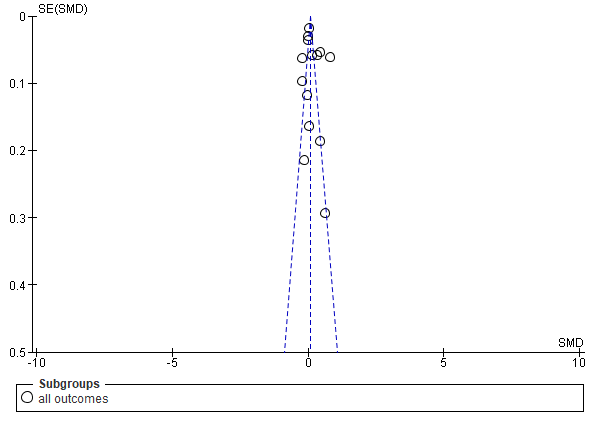
Connectedness**

**
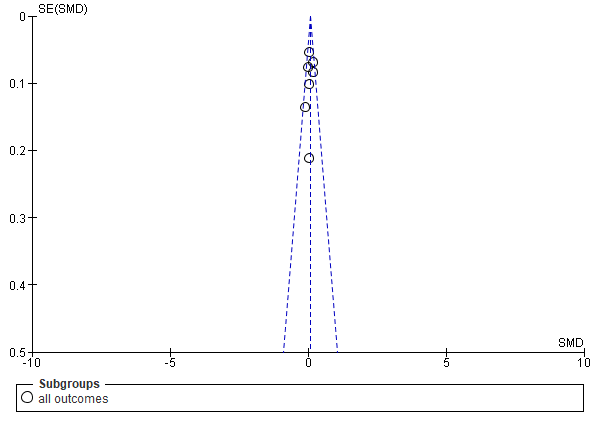
Hope and Optimism about the future**

**
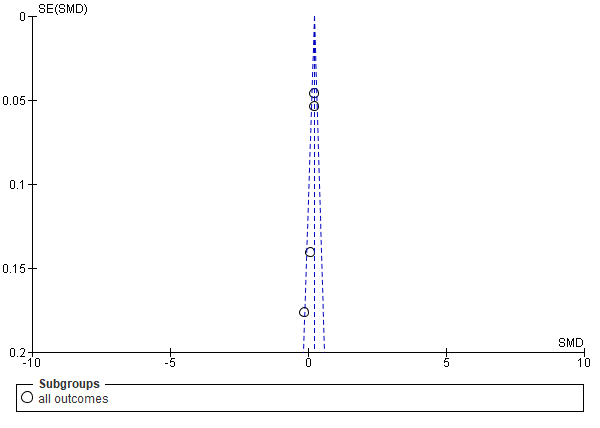
Identity**

**
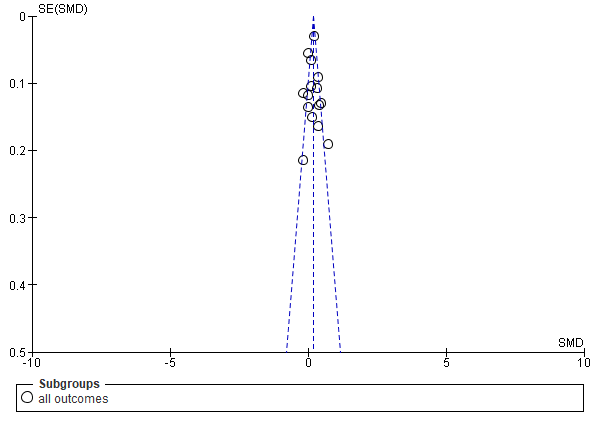
Meaning in life**

**
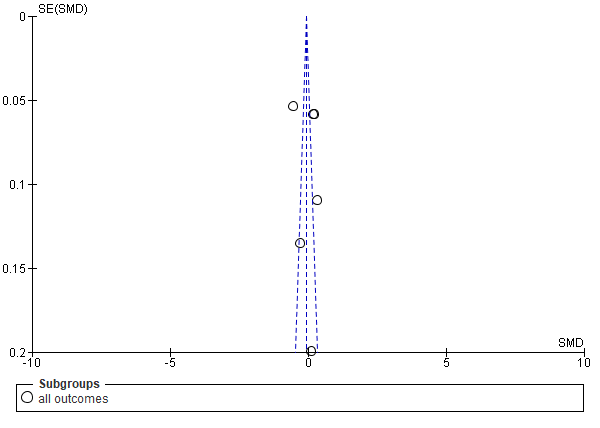
Empowerment**

**
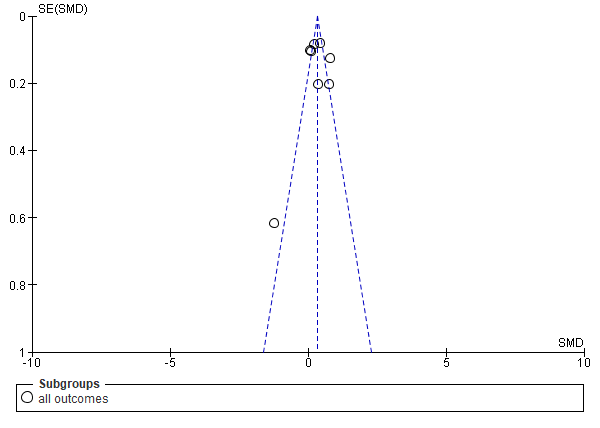
Overall personal recovery**

**Overall subjective quality of life**

**
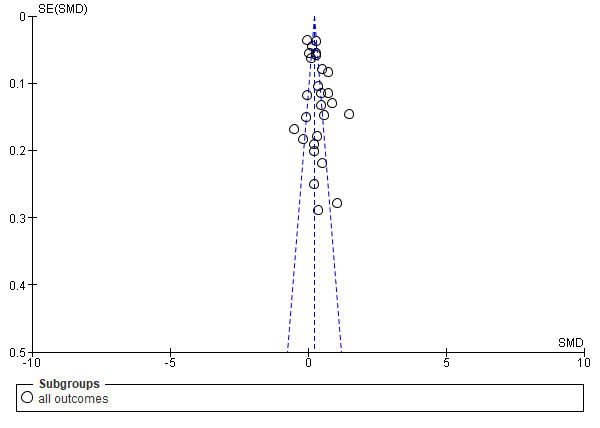
**

**Supplementary material 7.** Reference list of included studies

S1. Addington, J., & Addington, D. (2000). Neurocognitive and social functioning in schizophrenia: a 2.5 year follow-up study. *Schizophrenia research*, *44*(1), 47-56.

S2. Beaudoin, M., Hudon, A., Giguère, C. E., Potvin, S., & Dumais, A. (2022). Prediction of quality of life in schizophrenia using machine learning models on data from Clinical Antipsychotic Trials of Intervention Effectiveness (CATIE) schizophrenia trial. *Schizophrenia*, *8*(1), 29.

S3. Buonocore, M., Spangaro, M., Bechi, M., Baraldi, M. A., Cocchi, F., Guglielmino, C., ... & Cavallaro, R. (2018). Integrated cognitive remediation and standard rehabilitation therapy in patients of schizophrenia: persistence after 5 years. *Schizophrenia research*, *192*, 335-339.

S4. Chan, G. W. L., Ungvari, G. S., Shek, D. T. L., & Leung, J. P. (2003). Impact of deinstitutionalisation on the quality of life of Chinese patients with schizophrenia: A longitudinal pilot study. *Hong Kong Journal of Psychiatry*, *13*(4), 2-6.

S5. Chien, W. T., & Thompson, D. R. (2014). Effects of a mindfulness-based psychoeducation programme for Chinese patients with schizophrenia: 2-year follow-up. *The British Journal of Psychiatry*, *205*(1), 52-59.

S6. Chien, W. T., Chow, K. M., Chong, Y. Y., Bressington, D., Choi, K. C., & Chan, C. W. H. (2020). The role of five facets of mindfulness in a mindfulness-based psychoeducation intervention for people with recent-onset psychosis on mental and psychosocial health outcomes. *Frontiers in psychiatry*, *11*, 177.

S7. Conley, R. R., Ascher-Svanum, H., Zhu, B., Faries, D. E., & Kinon, B. J. (2007). The burden of depressive symptoms in the long-term treatment of patients with schizophrenia. *Schizophrenia research*, *90*(1-3), 186-197.

S8. Dellazizzo, L., Potvin, S., Phraxayavong, K., & Dumais, A. (2021). One-year randomized trial comparing virtual reality-assisted therapy to cognitive–behavioral therapy for patients with treatment-resistant schizophrenia. *npj Schizophrenia*, *7*(1), 9.

S9. Fernández-Modamio, M., Gil-Sanz, D., Arrieta-Rodríguez, M., Santacoloma-Cabero, I., Bengochea-Seco, R., González-Fraile, E., & Muñiz, J. (2021). A randomized study on the efficacy of the Social Cognition Training Program-brief version in a sample of patients with schizophrenia. *Psychiatric Rehabilitation Journal*, *44*(1), 1.

S10. Fowler, D., Hodgekins, J., Garety, P., Freeman, D., Kuipers, E., Dunn, G., ... & Bebbington, P. E. (2012). Negative cognition, depressed mood, and paranoia: a longitudinal pathway analysis using structural equation modeling. *Schizophrenia bulletin*, *38*(5), 1063-1073.

S11. Fowler, D., Hodgekins, J., French, P., Marshall, M., Freemantle, N., McCrone, P., ... & Birchwood, M. (2018). Social recovery therapy in combination with early intervention services for enhancement of social recovery in patients with first-episode psychosis (SUPEREDEN3): a single-blind, randomised controlled trial. *The Lancet Psychiatry*, *5*(1), 41-50.

S12. Galderisi, S., Rucci, P., Mucci, A., Rossi, A., Rocca, P., Bertolino, A., ... & Brasso, C. (2020). The interplay among psychopathology, personal resources, context‐related factors and real‐life functioning in schizophrenia: stability in relationships after 4 years and differences in network structure between recovered and non‐recovered patients. *World Psychiatry*, *19*(1), 81-91.

S13. Godin, O., Fond, G., Bulzacka, E., Schürhoff, F., Boyer, L., Myrtille, A., ... & Zinetti-Bertschy, A. (2019). Validation and refinement of the clinical staging model in a French cohort of outpatient with schizophrenia (FACE-SZ). *Progress in Neuro-Psychopharmacology and Biological Psychiatry*, *92*, 226-234.

S14. Górna, K., Jaracz, K., Rybakowski, F., & Rybakowski, J. (2008). Determinants of objective and subjective quality of life in first-time-admission schizophrenic patients in Poland: a longitudinal study. *Quality of Life Research*, *17*, 237-247.

S15. Gumley, A. I., Bradstreet, S., Ainsworth, J., Allan, S., Alvarez-Jimenez, M., Birchwood, M., ... & Gleeson, J. (2022). Digital smartphone intervention to recognise and manage early warning signs in schizophrenia to prevent relapse: the EMPOWER feasibility cluster RCT. *Health Technology Assessment (Winchester, England)*, *26*(27), 1.

S16. Hayhurst, K. P., Drake, R. J., Massie, J. A., Dunn, G., Barnes, T. R. E., Jones, P. B., & Lewis, S. W. (2014). Improved quality of life over one year is associated with improved adherence in patients with schizophrenia. *European Psychiatry*, *29*(3), 191-196.

S17. Heering, H. D., Janssens, M., Boyette, L. L., van Haren, N. E., & GROUP investigators. (2015). Remission criteria and functional outcome in patients with schizophrenia, a longitudinal study. *Australian & New Zealand Journal of Psychiatry*, *49*(3), 266-274.

S18. Ito, S., Nemoto, T., Tsujino, N., Ohmuro, N., Matsumoto, K., Matsuoka, H., ... & Mizuno, M. (2015). Differential impacts of duration of untreated psychosis (DUP) on cognitive function in first-episode schizophrenia according to mode of onset. *European Psychiatry*, *30*(8), 995-1001.

S19. Jørgensen, R., Zoffmann, V., Munk-Jørgensen, P., Buck, K. D., Jensen, S. O., Hansson, L., & Lysaker, P. H. (2015). Relationships over time of subjective and objective elements of recovery in persons with schizophreni. *Psychiatry Research*, *228*(1), 14-19.

S20. Fulford, D., Piskulic, D., Addington, J., Kane, J. M., Schooler, N. R., & Mueser, K. T. (2018). Prospective relationships between motivation and functioning in recovery after a first episode of schizophrenia. *Schizophrenia bulletin*, *44*(2), 369-377.

S21. Kane, J. M., Robinson, D. G., Schooler, N. R., Mueser, K. T., Penn, D. L., Rosenheck, R. A., ... & Heinssen, R. K. (2016). Comprehensive versus usual community care for first-episode psychosis: 2-year outcomes from the NIMH RAISE early treatment program. *American Journal of Psychiatry*, *173*(4), 362-372.

S22. Kelly, D. L., Weiner, E., Ball, M. P., McMahon, R. P., Carpenter, W. T., & Buchanan, R. W. (2009). Remission in schizophrenia: the relationship to baseline symptoms and changes in symptom domains during a one-year study. *Journal of Psychopharmacology*, *23*(4), 436-441.

S23. Kim, S. H., Hwang, S. S., Jung, H. Y., Kim, Y., Ahn, Y. M., Chung, I. W., & Kim, Y. S. (2019). Differences between self-reported and clinician-rated evaluations of 1-year changes in auditory verbal hallucinations among schizophrenia patients. *Progress in Neuro-Psychopharmacology and Biological Psychiatry*, *95*, 109671.

S24. Kumazaki, H., Kobayashi, H., Niimura, H., Kobayashi, Y., Ito, S., Nemoto, T., ... & Mizuno, M. (2012). Lower subjective quality of life and the development of social anxiety symptoms after the discharge of elderly patients with remitted schizophrenia: a 5-year longitudinal study. *Comprehensive Psychiatry*, *53*(7), 946-951.

S25. Lasser, R. A., Bossie, C. A., Gharabawi, G. M., & Kane, J. M. (2005). Remission in schizophrenia: results from a 1-year study of long-acting risperidone injection. *Schizophrenia Research*, *77*(2-3), 215-227.

S26. Lee, M. A., Cola, P., Jayathilake, K., & Meltzer, H. Y. (2023). Long-Term Outcome of Clozapine in Treatment-Resistant Schizophrenia. *Journal of Clinical Psychopharmacology*, *43*(3), 211-219.

S27. Litman, R., Naber, D., Anta, L., Martínez, J., Filts, Y., & Correll, C. U. (2023). Personal and Social Functioning and Health-Related Quality of Life in Patients with Schizophrenia Treated with the Long-Acting Injectable Antipsychotic Risperidone ISM. *Neuropsychiatric Disease and Treatment*, 219-232.

S28. Liu, C. C., Hsieh, M. H., Chien, Y. L., Liu, C. M., Lin, Y. T., Hwang, T. J., & Hwu, H. G. (2023). Guided antipsychotic reduction to reach minimum effective dose (GARMED) in patients with remitted psychosis: a 2-year randomized controlled trial with a naturalistic cohort. *Psychological Medicine*, 1-9.

S29. Lopez-Morinigo, J. D., Martínez, A. S. E., Barrigón, M. L., Escobedo-Aedo, P. J., Ruiz-Ruano, V. G., Sánchez-Alonso, S., ... & David, A. S. (2023). A pilot 1-year follow-up randomised controlled trial comparing metacognitive training to psychoeducation in schizophrenia: effects on insight. *Schizophrenia*, *9*(1), 7.

S30. Marino, L., Nossel, I., Choi, J. C., Nuechterlein, K., Wang, Y., Essock, S., ... & Dixon, L. (2015). The RAISE connection program for early psychosis: secondary outcomes and mediators and moderators of improvement. *The Journal of nervous and mental disease*, *203*(5), 365.

S31. McNeely, H. E., Letts, L., Martin, M. L., & Strong, S. (2023). Participants’ Evaluation and Outcomes following Integration of Self-Management Support into Outpatient Schizophrenia Case Management. *International Journal of Environmental Research and Public Health*, *20*(4), 3035.

S32. Moncrieff, J., Crellin, N., Stansfeld, J., Cooper, R., Marston, L., Freemantle, N., ... & Priebe, S. (2023). Antipsychotic dose reduction and discontinuation versus maintenance treatment in people with schizophrenia and other recurrent psychotic disorders in England (the RADAR trial): an open, parallel-group, randomised controlled trial. *The Lancet Psychiatry*, *10*(11), 848-859.

S33. Morrison, A. P., Law, H., Carter, L., Sellers, R., Emsley, R., Pyle, M., ... & Haddad, P. M. (2018). Antipsychotic drugs versus cognitive behavioural therapy versus a combination of both in people with psychosis: a randomised controlled pilot and feasibility study. *The Lancet Psychiatry*, *5*(5), 411-423.

S34. Na, E. J., Kang, N. I., Kim, M. Y., Cui, Y., Choi, H. E., Jung, A. J., & Chung, Y. C. (2016). Effects of community mental health service in subjects with early psychosis: One-year prospective follow up. *Community mental health journal*, *52*, 724-730.

S35. Neill, E., Rossell, S. L., Yolland, C., Meyer, D., Galletly, C., Harris, A., ... & Castle, D. J. (2022). N-acetylcysteine (NAC) in schizophrenia resistant to clozapine: a double-blind, randomized, placebo-controlled trial targeting negative symptoms. *Schizophrenia Bulletin*, *48*(6), 1263-1272.

S36. Ortega, L., Montalvo, I., Monseny, R., Burjales‐Martí, M. D., Martorell, L., Sanchez‐Gistau, V., ... & Labad, J. (2021). Perceived stress, social functioning and quality of life in first‐episode psychosis: A 1‐year follow‐up study. *Early Intervention in Psychiatry*, *15*(6), 1542-1550.

S37. Prouteau, A., Verdoux, H., Briand, C., Lesage, A., Lalonde, P., Nicole, L., ... & Stip, E. (2005). Cognitive predictors of psychosocial functioning outcome in schizophrenia: a follow-up study of subjects participating in a rehabilitation program. *Schizophrenia Research*, *77*(2-3), 343-353.

S38. Rowland, T., Birchwood, M., Singh, S., Freemantle, N., Everard, L., Jones, P., ... & Thompson, A. (2019). Short-term outcome of first episode delusional disorder in an early intervention population. *Schizophrenia Research*, *204*, 72-79.

S39. Luther, L., Fukui, S., Firmin, R. L., McGuire, A. B., White, D. A., Minor, K. S., & Salyers, M. P. (2015). Expectancies of success as a predictor of negative symptoms reduction over 18 months in individuals with schizophrenia. *Psychiatry research*, *229*(1-2), 505-510.

S40. Salyers, M. P., McGuire, A. B., Kukla, M., Fukui, S., Lysaker, P. H., & Mueser, K. T. (2014). A randomized controlled trial of illness management and recovery with an active control group. *Psychiatric services*, *65*(8), 1005-1011.

S41. Schmidt, S. J., Lange, M., Schöttle, D., Karow, A., Schimmelmann, B. G., & Lambert, M. (2018). Negative symptoms, anxiety, and depression as mechanisms of change of a 12-month trial of assertive community treatment as part of integrated care in patients with first-and multi-episode schizophrenia spectrum disorders (ACCESS I trial). *European archives of psychiatry and clinical neuroscience*, *268*, 593-602.

S42. Sikira, H., Janković, S., Slatina, M. S., Muhić, M., Sajun, S., Priebe, S., & Kulenović, A. D. (2021). The effectiveness of volunteer befriending for improving the quality of life of patients with schizophrenia in Bosnia and Herzegovina–an exploratory randomised controlled trial. *Epidemiology and Psychiatric Sciences*, *30*, e48.

S43. Tabo, A., Aydın, E., Yumrukçal, H., Yiğit, S., Uzun, U. E., & Karamustafalıoğlu, O. (2017). Longer duration of untreated psychosis hinders improvement in treatment of chronic schizophrenia: community based early intervention is an evidence based option. *Community Mental Health Journal*, *53*, 929-935.

S44. Usui, K., Kirihara, K., Tada, M., Fujioka, M., Koshiyama, D., Tani, M., ... & Kasai, K. (2022). The association between clinical symptoms and later subjective quality of life in individuals with ultra‐high risk for psychosis and recent‐onset psychotic disorder: A longitudinal investigation. *Psychiatry and Clinical Neurosciences*, *76*(11), 552-559.

S45. Veerman, S. R. T., Schulte, P. F. J., Deijen, J. B., & De Haan, L. (2017). Adjunctive memantine in clozapine-treated refractory schizophrenia: an open-label 1-year extension study. *Psychological Medicine*, *47*(2), 363-375.

S46. Wilson-d’Almeida, K., Karrow, A., Bralet, M. C., Bazin, N., Hardy-Baylé, M. C., & Falissard, B. (2013). In patients with schizophrenia, symptoms improvement can be uncorrelated with quality of life improvement. *European psychiatry*, *28*(3), 185-189.

S47. Wunderink, L., Sytema, S., Nienhuis, F. J., & Wiersma, D. (2009). Clinical recovery in first-episode psychosis. *Schizophrenia Bulletin*, *35*(2), 362-369.

S48. Drake, R. E., McHugo, G. J., Xie, H., Fox, M., Packard, J., & Helmstetter, B. (2006). Ten-year recovery outcomes for clients with co-occurring schizophrenia and substance use disorders. *Schizophrenia bulletin*, *32*(3), 464-473.

S49. Xie, H., McHugo, G. J., Helmstetter, B. S., & Drake, R. E. (2005). Three-year recovery outcomes for long-term patients with co-occurring schizophrenic and substance use disorders. *Schizophrenia research*, *75*(2-3), 337-348.

S50. Zäske, H., Linden, M., Degner, D., Jockers-Scherübl, M., Klingberg, S., Klosterkötter, J., ... & Gaebel, W. (2019). Stigma experiences and perceived stigma in patients with first-episode schizophrenia in the course of 1 year after their first in-patient treatment. *European archives of psychiatry and clinical neuroscience*, *269*, 459-468.
